# Virtual healthcare compared to hospital care for acute and post‐acute illness in adults: A systematic review and meta‐analysis of randomized controlled trials

**DOI:** 10.1002/bcp.70348

**Published:** 2025-11-29

**Authors:** Rana A. Malhis, Stuart E. Bond, Ahmed A. Sadeq, Rani Shatnawi, Barbara R. Conway, Syed Shahzad Hasan, Mamoon A. Aldeyab

**Affiliations:** ^1^ Department of Pharmacy, School of Applied Sciences University of Huddersfield Huddersfield UK; ^2^ Pharmacy Department Mid Yorkshire Hospitals NHS Trust Wakefield UK; ^3^ Department of Pharmacy Shaikh Shakhbout Medical City in Partnership with Mayo Clinic Abu Dhabi United Arab Emirates; ^4^ Allied Medical Sciences, Applied Medical Faculty Jordan University of Science and Technology Irbid Jordan; ^5^ Reading School of Pharmacy University of Reading Reading UK

**Keywords:** clinical pharmacology, Health policy, meta‐analysis, patient safety, pharmacy, statistics and study design, virtual healthcare

## Abstract

**Aim:**

To evaluate the clinical effectiveness, cost‐effectiveness, quality of life (QoL) and patient/caregiver satisfaction associated with VWs/HaH *vs*. traditional inpatient care in adults with acute or post‐acute illness.

**Methods:**

We conducted a systematic review and meta‐analysis of randomized controlled trials (RCTs), following PRISMA 2020 guidelines, registered with PROSPERO (CRD42024508457). Major databases were searched until October 2024. Primary outcomes were mortality, readmission, emergency attendance and length of stay. Secondary outcomes were quality of life (QoL), cost‐effectiveness, patient satisfaction and caregiver burden. Meta‐analyses employed random‐effects models; heterogeneity was assessed using the I^2^ statistic.

**Results:**

A total of 47 reports of RCTs (9749 patients) were included. Meta‐analyses showed no statistically significant differences in mortality over 1 to 12‐month periods (OR 0.82‐1.11, 95% CI 0.54‐1.43), readmission rates (OR 0.93‐1.16, 95% CI 0.80‐1.67) or emergency attendance rates (3‐month OR 0.86, 95% CI: 0.6–1.25). Narrative synthesis indicated VWs/HaH had higher patient satisfaction and potential cost savings. Quality of life outcomes were comparable, with some improvements in pain and emotional well‐being noted in home care settings.

**Conclusion:**

VWs/HaH models demonstrated non‐inferior clinical safety and cost‐effectiveness compared to inpatient care for select adult populations. High satisfaction and comparable clinical outcomes were observed. Findings support the continued, regulated integration of virtual care into routine practice. Future research should focus on service standardization, patient/caregiver satisfaction and support and specific economic evaluations.

## 1 INTRODUCTION

Healthcare systems are under increasing pressure due to ageing populations and rising rates of multi‐morbidity, associated with a higher risk of emergency attendance and hospital admissions^1^, ^2^ The COVID‐19 pandemic has intensified these challenges.^3^, ^4^ This has accelerated the search for alternatives to hospital inpatient care and led to the expansion of virtual healthcare models, including virtual wards and hospital‐at‐home services. Such models, which deliver acute or post‐acute care in patients' homes using a combination of in‐person visits and digital technologies, have emerged as an innovation in healthcare delivery.^5^

The terms ‘virtual ward’ and ‘hospital‐at‐home’ are used interchangeably; they describe “coordinated healthcare for an acute health condition that can be managed in the patient's home, which traditionally required a hospital stay”.^6^ They are delivered by a multidisciplinary team, including consultant medical practitioners, trained GPs, nurses and pharmacists. It is common to utilize digital platforms for remote monitoring and making timely clinical decisions. Two models are implemented: one is admission avoidance (AA), and the second is early supported discharge.^7^ Both approaches aim to improve patient outcomes, enhance healthcare quality and optimize staff experience while reducing hospital burden.^5^

The impact of virtual wards on different healthcare outcomes is still unclear.^5^ Many challenges and opportunities need to be addressed to fully realize their potential robust, up‐to‐date synthesis of the evidence is needed.

This systematic review and meta‐analysis aimed to evaluate the effectiveness of virtual healthcare models compared to traditional inpatient care for adults experiencing acute or post‐acute episodes of illness, informing healthcare policymakers on optimizing virtual ward implementations, as well as informing future research.

## 2 METHODS

### 2.1 Search strategy

In accordance with the Cochrane Handbook for Systematic Reviews of Interventions, this review was performed using the Preferred Reporting Items for Systematic Reviews and Meta‐Analyses (PRISMA) checklist. It was registered with the National Institute for Health Research (NIHR) PROSPERO (International Prospective Register of Systematic Reviews) (registration number is CRD 42024508457).^8^

A systematic search of four databases; Public/Publisher MEDLINE (PubMed), Cochrane Central Register of Controlled Trials, Cumulative Index to Nursing and Allied Health Literature (CINAHL) and Excerpta Medica Database (Embase) was undertaken independently by two reviewers (R.M. & R.S.) to identify RCTs comparing virtual healthcare to usual care for acute episodes, from inception to October 15, 2024. Table S1 summarizes the PICO approach (the inclusion and exclusion criteria), and the search strategy is outlined in Table S2 for each of the databases. In addition to database searching, we performed a forward citation analysis of all included studies using Google Scholar on October 20, 2024, using the same inclusion and exclusion criteria applied in the main search. The reference lists of included studies were also checked (backward citation).

### 2.2 Eligibility criteria

We included RCTs that compared admission to the virtual ward/hospital at home (including admission avoidance and early supported discharge) for the management of acute or post‐acute illness in the adult population, where the patient would otherwise be hospitalized, with hospital inpatient care. No restrictions were applied on language or geographical setting. For the included non‐English RCTs, we utilized translations available on the publisher's website. For a study to be included, the HaH intervention had to consist of at least one home visit by health care staff. Studies examining remote monitoring without at least one home visit by healthcare staff were excluded, as were studies of long‐term care or those that compared to any comparator other than hospital care. Disagreements regarding selection were resolved by discussion with a third reviewer (M.A.A.). Exclusion examples are listed in Table S3.

### 2.3 Data extraction

Two reviewers (R.M. and R.S. or A.S.) independently extracted data for five of the included RCTs to pilot the data extraction form, which included study characteristics, details of the intervention studied (type of virtual ward, inclusion criteria and outcome measures assessed) and key findings. The data were then extracted from all included RCTs. The primary clinical outcomes were mortality, readmission rates, emergency attendance and length of stay (LoS). We considered cost‐effectiveness, patient and caregiver satisfaction and quality of life as secondary outcomes.

Risk of bias was assessed using the Cochrane RoB 2 tool,^9^ evaluating five main domains (randomization, deviations from intended interventions, missing outcome data, outcome measurement and selection of the reported result). Two reviewers (R.M. and S.S.) independently assessed each RCT, and discrepancies were resolved through discussion or consultation with a third reviewer (M.A.A.).

Meta‐analyses were conducted using a random‐effect model to compensate for heterogeneity. Outcomes were pooled using odds ratios (OR) (for binary data) and mean differences (MD) (for continuous data) with 95% confidence intervals. Statistical heterogeneity was assessed using the I^2^ statistic, with I^2^ > 50% indicating moderate to high heterogeneity. A p‐value <0.05 and CI not crossing 1 (for binary data) or 0 (for continuous data) were considered statistically significant.

Outcomes analysed via meta‐analysis were mortality and readmission at various points in time, Length of Stay in care, emergency attendance within 3 months and patient satisfaction. For each performed meta‐analysis, the same outcome was measured using similar methods at the same point in time by at least three RCTs. Patient satisfaction was assessed at the end of the treatment period of the study. For outcomes that did not meet that criterion or were assessed by fewer than three RCTs, a narrative approach was used. Additionally, other outcomes were narratively synthesized due to diverse measurement approaches. For meta‐analysis that reached the ≥10‐study threshold, publication bias was assessed visually using funnel plot asymmetry and statistically using Egger's regression test. All statistical analyses and meta‐analyses were conducted using RevMan version 5.4 (Cochrane Collaboration, Copenhagen, Denmark) and MetaXL version 5.3 (https://www.epigear.com/index_files/metaxl.html).

## 3 RESULTS

**FIGURE 1:**
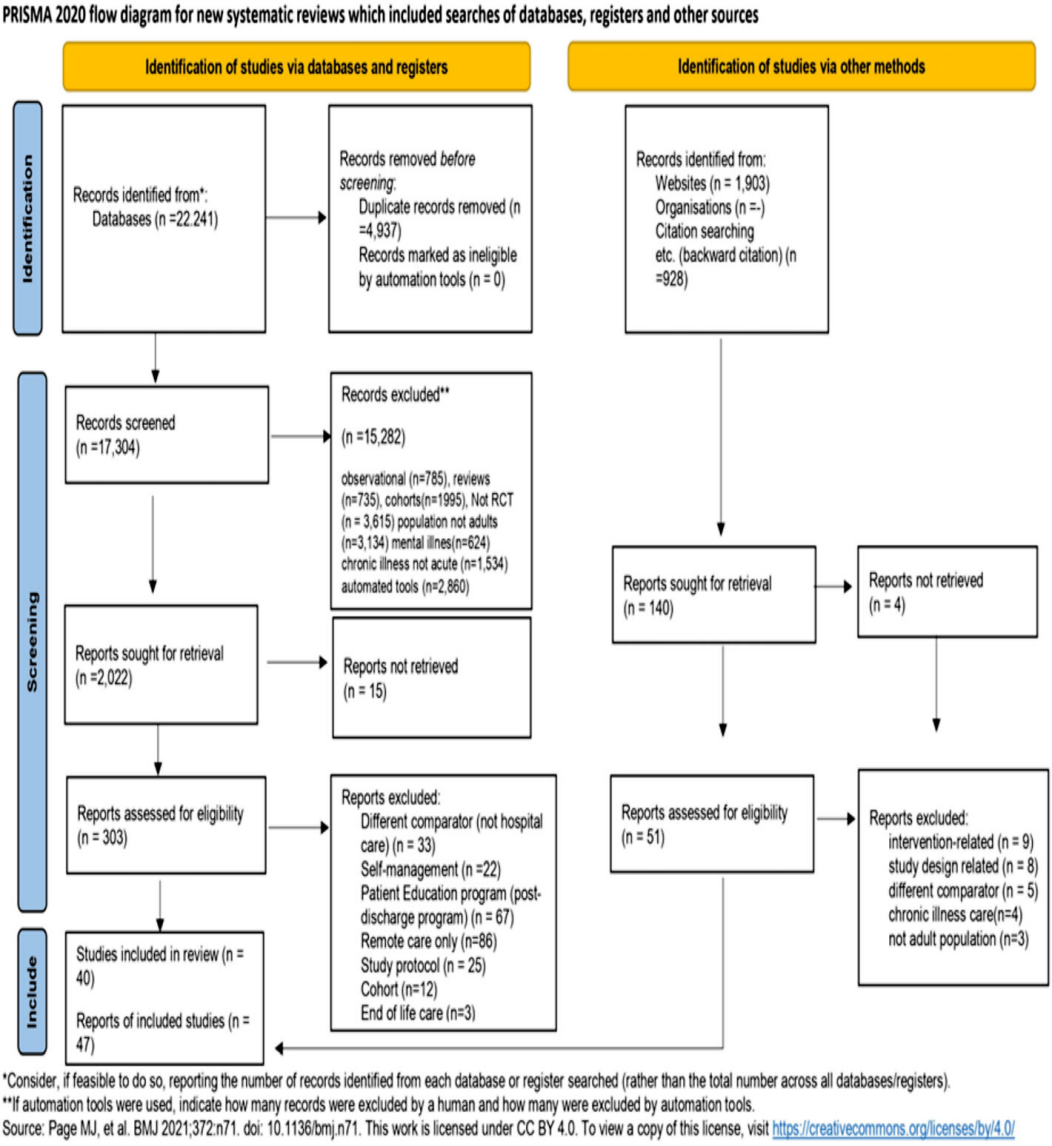
PRISMA (preferred reporting items for systematic reviews and meta‐analyses) flow diagram of the process of study selection. Source: Page MJ, et al. BMJ 2021;372:n71. Doi: 10.1136/bmj.n71. This work is licensed under CC BY 4.0. To view a copy of this licence, visit https://creativecommons.org/licenses/by/4.0/.

The search identified 22 241 records, of which 4937 were excluded as duplicates during title and abstract screening, resulting in 17 304 articles for screening. The PRISMA flow chart illustrates the selection, as shown in Figure 1. The main reasons for exclusion included chronic or mental illness, end‐of‐life care, or reasons related to the population, such as paediatric or pregnant participants (Table S3).

### 3.1 Summary of results

A total of 47 reports of 40 randomized controlled trials (RCTs) recruiting 9749 patients were included.^10^, ^11^, ^12^, ^13^, ^14^, ^15^, ^16^, ^17^, ^18^, ^19^, ^20^, ^21^, ^22^, ^23^, ^24^, ^25^, ^26^, ^27^, ^28^, ^29^, ^30^, ^31^, ^32^, ^33^, ^34^, ^35^, ^36^, ^37^, ^38^, ^39^, ^40^, ^41^, ^42^, ^43^, ^44^, ^45^, ^46^, ^47^, ^48^, ^49^, ^50^, ^51^, ^52^, ^53^, ^54^, ^55^, ^56^ The studies varied in terms of sample size, patient populations and specific interventions, but consistently highlighted key outcomes, including length of stay (OS), readmission rates, mortality and patient satisfaction. Of these, 26 focused on admission avoidance^10^, ^11^, ^12^, ^13^, ^17^, ^23^, ^25^, ^26^, ^28^, ^29^, ^30^, ^31^, ^32^, ^33^, ^37^, ^38^, ^39^, ^40^, ^44^, ^45^, ^46^, ^47^, ^49^, ^50^, ^51^, ^56^ and 20 on early supported discharge.^14^, ^15^, ^16^, ^18^, ^19^, ^20^, ^21^, ^22^, ^24^, ^27^, ^34^, ^35^, ^36^, ^41^, ^42^, ^43^, ^52^, ^53^, ^54^, ^55^ The majority of RCTs included “high‐risk for admission” patients, including those with frailty or mixed conditions (14 studies). Chronic Obstructive Pulmonary Disease (COPD) was studied in eight RCTs^13^, ^25^, ^26^, ^28^, ^31^, ^37^, ^39^, ^42^ Acute Decompensated Heart Failure (ADCHF) was studied in five,^12^, ^18^, ^23^, ^24^, ^25^ and infection as the main health condition was studied in three RCTs.^12^, ^17^, ^32^ Table 1 presents the main extraction table for the included studies.

**TABLE 1:** Extraction summary of characteristics and key findings of included RCTs.

Author, year	Sample size	Country	Care model	Main Health condition	Inclusion criteria	Intervention	Outcomes measured	Key findings
**Singh et al., 2022​​** ^10^	700 randomized to CGAHAH and 355 to hospital care.	UK	Admission Avoidance (AA)	Mixed conditions	Older adults presenting with acute health deterioration who are eligible for urgent healthcare.	Comprehensive Geriatric Assessment Hospital at Home (CGAHAH), which includes home visits by healthcare staff.	Health and social care costs length of hospital stay Total hours of unpaid help Mortality rates at 6 months follow‐up.	CGAHAH resulted in lower health and social care costs (−£2265) and societal costs (−£2840). CGAHAH utilized 62.76 fewer hours of unpaid help compared to the hospital group.
**Shepperd et al, 2021​​** ^11^	1055: 700 CGAHAH; 355 hospital admission.	UK	Admission Avoidance (AA)	Mixed conditions	Older people who were medically unwell and physiologically stable, referred for a hospital admission.	Comprehensive Geriatric Assessment Hospital at Home (CGAHAH), involves providing hospital‐level care at home for older adults who are medically unwell but physiologically stable, using a geriatrician‐led, multi‐disciplinary team approach	Living at home at six months. Secondary outcomes: New admission to long‐term residential care, mortality, health status, delirium and patient satisfaction.	Higher 1‐month readmissions for CGAHAH but at 6‐months comparable rates with no significant difference in mortality HaH reduced residential care admissions (5.7% *vs*. 8.7%; p < 0.001)
**Levine, D. M., et al. 2021​** ^12^​	91 eligible for interviews, 36 patients (19 home hospital and 17 control)	USA	Admission Avoidance	General medical conditions such as infections and heart failure exacerbations	participants were recruited from the emergency department, eligible based on geographic location, illness type and functional status (able to ambulate to a bedside commode)	Home hospital care, including nurse and physician home visits, intravenous medications, remote monitoring, video communication and point‐of‐care testing	Patient experiences with care teams, factors promoting healing (such as sleep and activity) and systems factors (logistics of admission and discharge).	Patients receiving home hospital care reported better experiences with their care team, highlighting greater continuity and availability of clinicians compared to those in traditional hospital settings, which contributed to a more personalized care experience
**Echevarria et al., 2018​** ^13^​	118 HAH *vs*. usual care (60 *vs*. 58)	UK	Admission Avoidance	COPD	Patients with ECOPD and a DECAF score of 0 or 1 with low‐risk exacerbations of COPD to hospital	Providing care at home under the supervision of a respiratory team, typically within 24 h of admission.		HaH cost‐effective (74% probability), shorter LOS (4 *vs*. 5 days; p = 0.001). Mean 90‐day costs were £1016 lower in HAH, readmission rates were similar. 90% of patients preferred HAH for subsequent ECOPD
**Dhalla et al., 2014​** ^14^	1923 (HaH: 961/Usual care: 962)	Canada	Early Supported Discharge (ESD)	High‐risk adults	Patients aged 18 years or older discharged from the general internal medicine ward of any of the four participating hospitals. being at high risk of readmission, as determined by a LACE score greater than 10 (LACE stands for length of stay, acuity of the admission, comorbidities and emergency department visits in the previous 6 months). resided within the Toronto Central Local Health Integration Network.	A post discharge virtual ward model where patients received care coordination and direct care from an interprofessional team through home visits, telephone calls and clinic visits for several weeks after hospital discharge	readmission to any hospital or death within 30 days of discharge. ‐ Readmission, death, emergency department visits and nursing home admission at 90 days, 6 months and 1 year of discharge. ‐	Virtual wards non‐inferior for readmissions (18.9% *vs*. 21.3%; p = 0.09).
**Utens, C.M. et al.,2014​** ^15^	115	Netherlands	Early Supported Discharge	COPD	Patients aged ≥40 years, competent to give informed consent, diagnosed with COPD, and hospitalized for COPD exacerbation	(Early Supported Discharge)	Informal caregiver strain, preference and satisfaction in hospital‐at‐home and usual hospital care for COPD exacerbations: Results of a randomized controlled tria	mean difference 0.36; p = 0.634); 71% caregivers preferred HaH
**Utens, C.M. et al.,** **2013​** ^16^	139: 69 usual hospital care; 70 ESD.	the Netherlands	Early Supported Discharge	(COPD) exacerbations	Patients aged ≥40 years, competent to give informed consent, diagnosed with COPD, and hospitalized for COPD exacerbation	Early assisted discharge	Patient satisfaction, preference for treatment place, clinical outcomes and economic evaluation	Similar satisfaction (71% *vs*. 70%), HaH patients felt less safe at night.
**Vianello et al., 2013​** ^17^	53 (26 hospital‐at‐home group and 27 hospitalized group).	Italy	Admission Avoidance	Neuromuscular Disease Patients with Respiratory Tract Infection	NMD patients suffering from severe respiratory tract infections; living within the geographic area covered by the district nurse service	patients with severe respiratory tract infections, utilizing continuous non‐invasive ventilation (NIV), assisted cough techniques and regular visits from healthcare professionals, including pulmonologists and district nurses	Need for hospitalization, treatment failure, time to recovery, mortality within three months and healthcare costs	HaH reduced hospitalizations (8 *vs*. 27; p < 0.05), lower costs (€542 *vs*. €8890).
**SOLETO et al. 2013** ^18^​​	71 completed the follow‐up	Spain	Early Supported Discharge	Acute decompens ation of chronic heart failure (ADCHF)	Patients aged 65 years or older with a history of heart failure for at least one year, NYHA Class II or III, requiring admission for decompensation and without severe comorbidities or recent hospitalizations	Hospital‐at‐Home (HaH) service, where patients received care at home with daily visits from a nurse and doctor	health‐related quality of life (HRQOL) assessed using the Barthel Index, EQ‐5D, SF‐36, and Minnesota Living with Heart Failure Questionnaire (MLHFQ) at multiple time points	HaH shortened LOS (9.2 *vs*. 12.2 days; p < 0.05), fewer relapses (0 *vs*. 5; p < 0.05).
**Goossens et al., 2013​** ^19^	139; 69 usual hospital care; 70 ESD	Netherlands	Early Supported Discharge	COPD	Age 40 years or older, competent for informed consent, smoking history of 10 or more pack‐years, improved physical and respiratory complaints, able to visit the toilet independently	Early assisted discharge after 3 days of hospital treatment, followed by homecare from community nurses	Change in Clinical COPD Questionnaire (CCQ) score, proportion of patients with clinically relevant improvement in CCQ score, quality‐adjusted life‐years (QALYs	Early discharge saved healthcare costs (from a health care perspective but not from a societal perspective), no mortality difference (p > 0.05). Or readmission, CCQ score was higher in the usual hospital care group after 7 days
**Utens et al, (2012)​** ^20^	139 (Early discharge: 70/Usual care: 69)	UK	Early Supported Discharge	COPD	Age ≥ 40 years, competent to give written informed consent, diagnosed with COPD (at least GOLD stage I and 10 pack years of smoking), hospitalization for COPD exacerbation.	Early assisted discharge with home care provided by community nurses.	Health status changes (CCQ scores), treatment failures, readmissions, mortality and health‐related quality of life (HRQL).	Similar readmissions (17 *vs*. 17), no mortality difference.
**Hendricks et al., 2011​** ^21^​	92 (57 hospital care group;35 home care group)	US	Early Supported Discharge	Fever and Neutropenia	Patients with chemotherapy‐related fever (≥100.5 °F) and neutropenia (absolute neutrophil count ≤500/μL), considered low risk for medical instability and able to use emergency medical assistance	early discharge to home care with intravenous (IV) antibiotics for patients with chemotherapy‐related febrile neutropenia, while ensuring daily monitoring by healthcare professionals	Direct medical costs, indirect costs (out‐of‐pocket expenses, caregiver time) and clinical outcomes (e.g., readmission rates, major medical complications)	HaH reduced costs (10977*vs*.10977vs.16341; p < 0.01), similar complications (9% vs. 8%).
**Talcott et al., 2011​** ^22^	117; (66 in hospital care/47 ESD	United States	Early Supported Discharge	febrile neutropenia	Adult outpatients with post‐chemotherapy fever (≥100.5 °F) and neutropenia (absolute neutrophil count < 500/μL) after at least 24‐hour inpatient observation, with no indications for hospitalization other than fever and neutropenia	early discharge to receive identical antibiotic treatment at home	the occurrence of any serious medical complication requiring urgent medical attention, along with patient‐reported quality of life and economic costs	(QOL): HaH reduced pain scores (−13.1 *vs*. +2.72; p = 0.01): rate of major medical complications was similar
**Tibaldi et al., 2009​** ^23^	101; GHHS: 48, GMW: 53	Italy	Admission Avoidance (AA)	Acute Decompensated Heart Failure	Patients aged 65 years or older with a confirmed diagnosis of heart failure, in New York Heart Association (NYHA) functional class II or III, and presenting with decompensated chronic heart failure symptoms.	a physician‐led Geriatric Home Hospitalization Service (GHHS) that provided hospital‐level care at home for elderly patients with acute decompensation of chronic heart failure (CHF).	Mortality, subsequent hospital admissions, functional status (Barthel Index), depression (Geriatric Depression Scale), cognitive status (Mini‐Mental State Examination), quality of life (Nottingham Health Profile), caregiver stress.	HaH improved depression scores (−1.48 *vs*. −0.86; p = 0.02) and delayed readmissions (84.3 *vs*. 69.8 days; p = 0.02).
**Mendoza et al., 2009​** ^24^	80: 71 completed it (37 in HaH and 34 in IHC).	Spain	Admission Avoidance (AA)	Acute Decompensated Heart Failure	Diagnosed with chronic heart failure with diastolic or systolic left ventricular dysfunction; deterioration of HF for ≥ 3 days with symptoms such as increasing dyspnoea, orthopnoea, weight gain, peripheral oedema or abdominal swelling.	Providing hospital‐level care at home (HaH) for patients with scheduled and urgent visits from an internal medicine specialist and a nurse, along with necessary medical care such as laboratory tests and ECGs	mortality, readmission due to HF or other cardiovascular events, changes in functional status (Barthel index), health‐related quality of life (SF‐36) and healthcare costs associated with each type of care.	HaH lowered initial costs (€2541 *vs*. €4502; p < 0.001), similar mortality.
**Patel et al. (2008)​** ^25^	31 (13 home group, 18 conventional care group)	Sweden	Admission Avoidance (AA)	Acute Decompensated Heart Failure	Patients with acute exacerbations of COPD requiring hospitalization, appropriate care supervision at home and informed consent.	Providing home care (HC) under the direction of a specialist nurse for patients with worsening chronic heart failure, including daily or every other day home visits and follow‐up care after an initial hospitalization	Clinical signs and symptoms, health‐related quality of life (HRQL), healthcare costs and patient satisfaction.	HaH feasible for CHF, reduced costs, no adverse events.
**Aimonino Ricauda et al, (2008)​** ^26^​	104 elderly (52 in Geriatric Home Hospitalization;52 in General Medical Ward)	Italy	Admission Avoidance (AA)	COPD		a Geriatric Home Hospitalization Service (GHHS) providing hospital‐level care at home for elderly patients with COPD exacerbations, using a multidisciplinary team for comprehensive treatment and monitoring	Hospital readmission rates, mortality, quality of life, depression scores, Activities of Daily Living scores, and patient satisfaction	No significant caregiver stress difference (25.4 *vs*. 17.1; p = 0.37).
**Mohammad et al., 2008​​** ^27^	234	Canada	Early Supported Discharge	Osteoarthritis, Inflammatory arthritis or Osteonecrosis	Age: Over 18 years Unilateral hip or knee replacement for specific health conditions Residents of the city of study Fluent in English Provided informed consent	Home‐based rehabilitation *vs*. Inpatient rehabilitation	WOMAC function score,Short Form‐36, Patient satisfaction with Hip and Knee Satisfaction Scale	HaH can significantly reduce the cost of care delivery following total hip or knee replacement, without compromising the quality of care or patient satisfaction
**Jaume Puig‐Junoy et al., 2007​** ^28^​	180 (Home care 103; hospital 77)	Spain	Admission Avoidance	COPD	patients admitted to the emergency room due to COPD exacerbation without criteria for imperative hospitalization as per British Thoracic Society guideline	Home hospitalization (HH) where a specialized respiratory nurse delivered integrated care at home	Direct healthcare costs, in‐patient hospital readmissions, emergency room visits, nurse home visits and quality of life	The average direct cost per patient was significantly lower for the home hospitalization group (€1154) compared to the conventional hospitalization group (€1964), resulting in a difference of €810 in favour of home hospitalization
**Dee A Richards et al., 2005​** ^29^​	49: 24 home treatment, 25 hospital treatment	New Zealand	Admission Avoidance	Community Acquired Pneumonia	Patients with community‐acquired pneumonia (CAP) and a CURB‐65 score of 0‐2, indicating low mortality risk.	Managing mild to moderately severe community‐acquired pneumonia (CAP) at home through the Extended Care @ Hme (EC@H) program, which provided daily GP visits, twice‐daily nurse visits and home‐based IV antibiotic services.	Primary: Duration to discharge, duration of IV and oral antibiotics, self‐rated symptom severity, general functioning (SF‐12 scale). Secondary: Complications and patient satisfaction.	HaH delayed discharge (median 4 *vs*. 2 days; p = 0.004), similar complications.
**Harris et al. (2005)** ^30^​	285 (HAH) Care: 143 Early Discharge: 104 Admission Prevention: 39 Hospital Care: 142 Early Discharge: 105 Admission Prevention: 37	New Zealand	Admission Avoidance (AA)	high risk adults	Patients aged 55 years or over with an acute medical problem, not booked for major surgery within 36 days, and suitable living arrangements.	Service that provides intensive clinical support to patients in their own homes instead of being admitted to a hospital.	Readmission rates, additional hospital days and mortality within 60 days of initial admission	HaH shortened LOS (3.2 *vs*. 6.1 days; p < 0.05), similar readmissions (29.3% *vs*. 30%).
**Lobato, et al., 2005​​** ^31^	40 (20 Home hospital group/20 Hospital group)	**Spain**	Admission Avoidance (AA)	**COPD**	Number of calls for consultation or medical assistance, number of relapses at 1 month follow‐up, extent of smoking cessation, length of 20hospitalizati21on, and therapeutic failures	COPD patients were treated at home after a short hospital stay, with regular visits from a pneumologist and nursing staff to monitor their condition and provide care	Mortality, hospital readmission rates, emergency room visits, quality of life, disease knowledge, self‐management, patient satisfaction and cost‐effectiveness	HaH improved quality of life, fewer ER visits, similar readmissions (30% *vs*. 29.3%).
**Caplan et al, 2005​​** ^33^	100 (GHHS: 53/GMW: 47)	**Australia**	Admission Avoidance	**high risk adults**	patients older than 65 years, but younger patients were also accepted conditions amenable to home treatment, such as pneumonia, urinary tract infections, cellulitis. excluded if they had evidence of shock, required oxygen, were judged too unwell by the study team, had no available carer, lived outside the local area	Effect of Hospital in the Home Treatment on Physical and Cognitive Function: A Randomized Controlled Trial	Barthel index, Instrumental Activities of Daily Living (IADL) index, and Mental Status Questionnaire (MSQ) scores, assessed at both admission and discharge	HaH shortened LOS (9.2 *vs*. 12.2 days; p < 0.05), fewer relapses (0 *vs*. 5; p < 0.05).
**Corwin, et al., 2005​** ^32^	200 (101 Home/99 Hospital)	New Zealand	Admission Avoidance (AA)	cellulitis	Patients have clinical signs of cellulitis assessed as requiring intravenous antibiotic treatment needed to be 16 years or older and mentally competent to give informed consent. They had to have a telephone at home and a caregiver nearby. Patients were required to be currently resident in the Christchurch metropolitan area	Administering intravenous cephazolin to patients with cellulitis at home, with daily visits from general practitioners and community care nursing staff to monitor progress and administer treatment.	Days to no advancement of cellulitis. Total number of days on intravenous antibiotics. Total number of days on oral antibiotics. Days in hospital or under home care. questionnaires. Patients' satisfaction	No difference in cellulitis resolution (1.5 *vs*. 1.49 days; p = 0.23), 90% patient preference for HaH.
**Booth, et al., 2004** ^34^​​	97 (32 conventional care, 65 early discharge)	UK	Early Supported Discharge	Heart Disease	Patients required first‐time isolated coronary artery bypass grafting (CABG). Patients had a carer available to stay with them. Patients lived within the travel area of the homecare team	Planned early discharge homecare program for patients undergoing coronary artery bypass grafting (CABG), which includes enhanced preoperative preparation and specialist homecare after surgery.	Length of hospital stay, in‐hospital clinical events, total costs, readmission rates and quality of life at 12 weeks.	HaH reduced costs (£6127 *vs*. £6381) and readmissions (17% *vs*. 34%; p = 0.19).
**Cunliffe et al. 2004​** ^35^	370 (185 in each arm)	UK	Early Supported Discharge	Activity limitation and rehabilitation needs in older people	patients aged 65 or above, residing within the Nottingham Health Authority boundary, medically fit for discharge and having rehabilitation needs that could be met at home	Early Discharge and Rehabilitation Service (EDRS) offering home‐based care for up to 4 weeks, staffed by a multidisciplinary team	Health‐related quality of life (HRQOL) assessed using the Barthel Index, EQ‐5D, SF‐36 and Minnesota Living with Heart Failure Questionnaire (MLHFQ) at multiple time points	HaH reduced readmissions (42% *vs*. 87%; p = 0.001), improved depression scores (−3.1 *vs*. −0.7; p = 0.00). EDRS group had fewer hospital days at 3 months, better Barthel scores, and improved psychological well‐being.
**Donnelly et al., 2004​​** ^36^	113 patients randomized into the trial (59 to the CST service and 54 to usual inpatient rehabilitation)	Belfast, Northern Ireland	Early Supported Discharge	stroke	Patients were eligible if they experienced a stroke within the last 4 weeks, had the potential to benefit from rehabilitation, were not residents of a nursing home, and had no pre‐existing disabilities that would hinder rehabilitatio	community‐based multidisciplinary stroke team (CST) that provided rehabilitation services at home	Hospital stay duration, functioning (Barthel Index, Nottingham ADL), quality of life (SF‐36), patient and carer satisfaction and caregiver strain	CST group reported significantly higher satisfaction with services (P = 0.01) compared to the hospital rehabilitation group. overall costs for the CST group were lower, although not statistically significant
**Hernandez et al.2003​** ^37^	222 patients (Home Hospitalization: 121/Conventional Care: 101)	**Spain**	Admission Avoidance (AA)	**COPD**	Patients with exacerbated COPD who were candidates for the study after screening.	Comprehensive home care managed by a specialized team, focusing on early discharge from the hospital and ongoing support at home.	Caregiver burden measured using the Zarit Burden Interview‐12 (ZBI‐12) at admission and within 30 days of discharge	No caregiver burden difference (ZBI‐12: 9.5 *vs*. 8.0; p = 0.98), high HaH preference.
**Wilson et al., 2002​** ^38^​	199 (102HaH; 97hopiital) for the quantitative analyis: (83) HaH; 48:Hospital35	**UK**	Admission Avoidance (AA)	**Mixed conditions**	Patients referred to the “hospital at home” scheme with an acute condition, meeting the admission requirements for both care settings.	Specialist nurses provided treatment and monitored patients in their homes as an alternative to inpatient admission.	Carer strain, patient satisfaction	Similar carer strain, higher HaH satisfaction.
**Ojoo, et al., 2002​** ^39^​	60 (HaH: 30/Control: 30)	UK	Admission Avoidance (AA)	COPD	Patients older than 65 years requiring admission for acute or subacute infections, assessed by medical or surgical teams. Exclusions included evidence of shock, need for oxygen, unsuitable home conditions,	Hospital at Home (HaH) care for patients with acute exacerbations COPD	Patient and carer preferences for site of care, satisfaction with care received, clinical outcomes (FEV1, FVC, symptom scores), readmission rates.	HaH preferred by patients/carers, improved IADL scores (p < 0.05).
**Nicholson et al., 2001​** ^40^	25 patients were randomized from a total of 168 candidates, representing 15% of hospital admission	Australia	Admission Avoidance	Acute Chronic Obstructive Pulmonary Disease (COPD)	Age > 45 years, documented diagnosis of COPD, current or ex‐smoker, FEV1 < 60% predicted, admission requested by general practice or considered necessary, willing to give informed consent and having a telephone at home	Integrated home‐based care model compared to traditional inpatient care	Cost per separation, lung function improvement, anxiety levels, and patient satisfaction	home care costs per separation were significantly lower ($745) compared to hospital care ($2543), with no significant difference in clinical outcomes or patient satisfaction
**GUNNELL et al., 2000​** ^41^​	241 of which had 133 identified main carers (93 hospital‐at‐home/40 hospital care)	UK	Early Supported Discharge	mixed conditions	Patients presenting with exacerbations of COPD, excluding those with impaired consciousness, acute confusion, significant radiographic changes or arterial pH < 7.35,	an early discharge hospital‐at‐home scheme where patients were discharged from the hospital to receive care at home, supported by a dedicated hospital‐at‐home team.	Modified 12‐item Carer Strain Index, COOP‐WONCA charts and EuroQol EQ‐5D at 4 weeks and 3 months post‐randomization.	HaH reduced costs (£877 *vs*. £1753), longer discharge time (7 *vs*. 5 days; p < 0.01).
**E Skwarska, et. al 2000​** ^42^​	184 (122 home support/62 hospital admission)	Scotland	Early Supported Discharge	COPD	Adults >18 years with FEV1/FVC ratio <70% and FEV1 reversibility to salbutamol <15%	Home supported discharge program for patients with exacerbations of COPD, allowing them to receive appropriate treatment and follow‐up care at home.	Readmission rates, quality of life (using the Chronic Respiratory Questionnaire), patient satisfaction and health service costs.	HaH improved FEV1 (0.16 L *vs*. 0.06 L; NS), high satisfaction (96.3% preferred HaH).
**Cotton et al., 2000​** ^43^​	81 (41 early discharge/40 conventional care).	UK	Early Supported Discharge	COPD	Patients admitted as emergencies with a diagnosis of COPD, excluding those with other medical conditions requiring inpatient management or acidotic respiratory failure.	Early discharge of patients with exacerbations of COPD, allowing them to continue treatment at home with support from respiratory nurses, ideally within three days of admission	Direct healthcare costs, clinical outcomes, quality of life and readmission rates	HaH reduced ER readmissions (9.6% *vs*. 22.3%) and costs (€1154 *vs*. €1964).
**Board et al., 2000​** ^44^​	100: 51 HaH; 49 Hospital	**Australia**	Admission Avoidance (AA	**high risk adults**		A randomized controlled trial of the costs of hospital as compared with hospital in the home for acute medical patients	Readmission rate in 28 days, mortality, cost per separation, patient satisfaction	no outcome differences (readmissions: 37% *vs*. 34%; mortality: 9% *vs*. 8%). HITH group had significantly lower costs per separation ($1764) compared to the control group ($3614) with no significant difference in clinical outcomes and higher satisfaction for HITH
**Davies, et al., 2000​** ^45^​	150 (100 home care/50 hospital care	UK	Admission Avoidance (AA)	COPD	Patients with exacerbations of COPD who identified a main helper (carer) before randomization	Providing “hospital at home” care specialist nurses administered treatment and monitored patients in their homes	Readmission rates at two weeks and three months, changes in forced expiratory volume in one second (FEV1), and mortality rates	There was no significant difference in FEV1 improvement after bronchodilator use between home care and hospital care groups at 2 weeks and 3 months. Readmission rates and mortality rates at three months were similar.
**Wilson et al., 1999​** ^50^​	199 (HaH: 100/Hospital: 97)	**UK**	Admission Avoidance (AA)	**mixed conditions**	Patients referred to the “hospital at home” scheme with an acute condition, meeting the admission requirements for both care settings.	Mortality, health status (using scales such as the sickness impact profile, Barthel index, Philadelphia geriatric morale scale, and EuroQol), and process metrics like length of stay and readmission rates.	Rate of care consumption, costs of professional and informal care, wound and drain complications, duration and amount of seromas, number of axillary drainage aspirations	HaH cost‐saving (*1764 vs*.1764*vs*.3614; p < 0.0001), no outcome differences.
**Caplan et al., 1999** ^51^​	100	**Australia**	Admission Avoidance	**HIGH RISK Patients**	Patients assessed in the emergency department as requiring admission to hospital	Providing hospital‐level care at home for patients, where they received treatments such as intravenous antibiotics and blood transfusions administered by a community outreach team within 24 h of diagnosis. Patients were monitored through regular home visits by study nurses, ensuring they received appropriate medical care in a familiar environment	Mortality, length of stay, quality of life (using EQ‐5D and COOP‐WONCA charts) and carer strain index.	HaH improved quality of life for hip patients, unsuitable for knees.
**Shepperd et al., 1998​** ^53^​	241	**UK**	Early Supported Discharge	**mixed conditions**	Patients recovering from hip and knee replacements, hysterectomy, elderly medical patients and those with chronic obstructive airways disease1.	Scheme provides home‐based nursing and rehabilitation services for patients who are medically stable and suitable for early discharge	Lengh of stay: Dartmouth COOP chart to measure patients' general health status; SF‐36	No major differences in outcome for any of the patient groups except that those recovering from hip replacement reported a significantly greater improvement in quality of life with hospital at home. HaH did not seem suitable for patients recovering from a knee replacement.
**Bonnema et al., 1998​** ^52^	125 (Short stay: 62/Long stay: 63)	Netherlands,	Early Supported Discharge	CANCER	Patients with stage I or II breast cancer who had surgery for a primary tumour in the breast and axillary dissection	Short postoperative hospital stays for breast cancer patients, with discharge on the morning of the fourth postoperative day while the drain was still in place. Community nursing care was provided, including scheduled home visits and 24‐hour telephone support, to ensure continuity of care after early discharge	Geriatric complications (confusion, falls, urinary incontinence or retention, faecal incontinence or constipation, phlebitis and pressure areas), patient/carer satisfaction, adverse events and death.	HaH reduced complications (0% *vs*. 20.4% confusion; p < 0.05). Early discharge post‐breast cancer surgery did not increase physical/psychological illness rates (p > 0.05).
**Jones et al., 1999​** ^31^​	199	**UK**	Admission Avoidance	**mixed conditions**	Patients assessed as being suitable for admission to hospital at home for acute care.	“Hospital at Home” scheme, which provides an alternative to traditional hospital inpatient care by delivering acute care to patients in their own homes	Costs to NHS, social services, patients and families during the initial episode of treatment and the three months after admission.	HaH saved $1320/patient, increased home care use.
**Makela et al., 2020​​** ^46^	34 older people (15 received hospital at home (HAH) care, 19 received hospital inpatient care)	United Kingdom	Admission Avoidance	Varied; included falls, delirium, exacerbation of chronic obstructive pulmonary disease, back pain, leg pain	Older individuals (not in end‐of‐life care), ability to participate (medically stable), variability in characteristics affecting illness management	Geriatrician‐led admission avoidance hospital at home (HAH)	Experiences of patients and caregivers, roles in managing acute health events, integration of acute and chronic healthcare, adaptive capacity	The ongoing modifications made by patients and their families promote the safety and quality of medical care.
**Moss, Levine et al., (2024)​** ^47^	91; 42 had caregivers (22 in home group, 11 in control group).	**USA**	Admission Avoidance (AA)	**high‐risk adults**	Patients with qualifying illness (e.g., infection, heart failure), adequate functional status (able to ambulate) and severity requiring admission but at low risk of needing intensive care.	Providing hospital‐level care at home, including twice daily nurse visits, once daily physician visits, in‐home diagnostics and continuous monitoring for patients with acute illnesses.	Health outcomes (physical and mental function, self‐rated recovery, health status via SF‐36), satisfaction surveys, Carer Strain Index, costs of care.	HaH increased satisfaction (93 *vs*. 87 patients rated “excellent”), higher costs (*NZ*6,524 *vs. NZ*3,525).
**Richards et al., 1998​** ^54^	241	UK	Early Supported Discharge	Mixed conditions: hip replacement, hysterectomy, elderly medical patients and those with COPD	Adult patients on an acute hospital ward, residing within the catchment area, with a positive rehabilitative outcome expected, appropriate home circumstances, and general practitioner acceptance of clinical responsibility	“hospital at home” scheme, providing nursing and rehabilitation services as an alternative to inpatient hospital care	Mortality, quality of life, physical functioning, patient satisfaction, length of stay, hospital readmissions, carer strain and patients' and carers' preferred form of care	Similar satisfaction (71% *vs*. 70%), HaH patients felt less safe at night. No significant differences in mortality, quality of life and physical functioning
**Bunderd et al., 1998** ^55^	100 women (Early discharge: 50/Standard: 50)	UK	Early Supported Discharge	Breast Cancer	Physical illness (infection, seroma formation, shoulder movement) and psychological illness (checklist of concerns, Rotterdam symptom questionnaire, hospital anxiety and depression scale) preoperatively and at one and three months postoperatively.	early discharge from the hospital two days after breast cancer surgery, with the axillary drain still in place. Specialist breast nurses played a crucial role by telephoning the patients daily and visiting them every other day to monitor their condition and provide support.	Length of stay, readmission time to drain removal, wound pain infection rate, shoulder movement, psychological outcomes.	HaH reduced pain scores (−13.1 *vs*. +2.72; p = 0.01), similar complications (9% *vs*. 8%). Women discharged early had greater shoulder movement and less wound pain three months after surgery compared with women given standard management.
**J. G. Zimmer et al., 1985​​** ^56^	167 (85 team care; 82 control group)	USA	Admission Avoidance (AA)	FRAILTY	Home‐bound patients wishing to stay at home, with significant illness requiring medical care, having a family member or friend to help and willing to participate in the research.	Hospital at Home” which provides an alternative to traditional hospital inpatient care	Health care system utilization. Patient health status. Patient satisfaction with health care. Caretaker satisfaction with health care. Functional changes in patients.‐ Mortality.	HaH reduced hospital utilization, higher satisfaction.

### 3.2 Quality assessment/risk of bias

**FIGURE 2:**
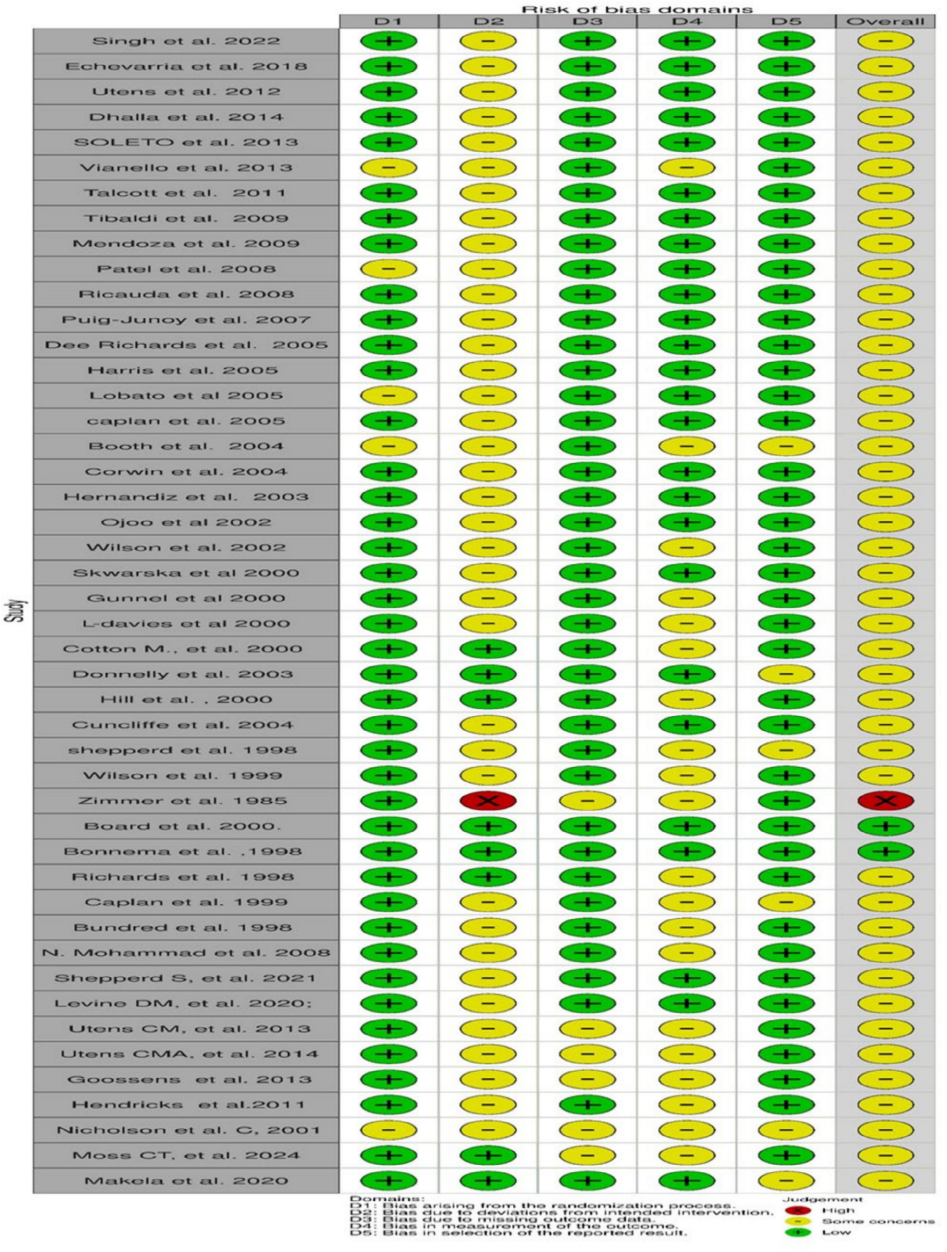
Quality assessment for RCTs' risk of bias using Rob2 tool. Using robvis tool for visualization generation: McGuinness, LA, Higgins, JPT. Risk‐of‐bias VISualization (robvis): an R package and shiny web app for visualizing risk‐of‐bias assessments. Res syn meth. 2020; 1‐7. https://doi.org/10.1002/jrsm.1411.

We evaluated the quality of the included RCTs using the six criteria recommended by the Cochrane Collaboration tool, risk of bias (ROB) II for RCTs.^9^ Risk of bias appraisal is presented in summary Figure 2, showing three levels of bias: **low risk of bias, some concerns or high risk of bias**.

Most RCTs had a moderate risk of bias. While randomization bias was minimal in most RCTs, performance bias was a concern as participants were logically aware of their assigned arm (home and hospital settings). Through matching baseline characteristics, outcome measurements and using the appropriate statistical adjustments, the overall risk was minimized as this type of bias was also unavoidable due to the trial context in the included RCTs.

Most meta‐analyses in this review did not reach the ≥10‐study threshold. Consistent with guidance, formal tests (e.g., Egger/Begg/Harbord/Peters) would be underpowered and potentially misleading with a small number of studies and high heterogeneity. For transparency, we generated funnel plots for outcomes with the largest numbers of included studies (e.g. readmission at 3 months with eight studies) and provided this in the Supplement (Figure S1). These plots are presented as exploratory only and interpreted cautiously.

## 4 RESULTS OF META‐ANALYSIS

### 4.1 Mortality and readmission

A total of 23 RCTs were included in the meta‐analysis for the mortality outcome.^11^, ^13^, ^14^, ^17^, ^18^, ^20^, ^21^, ^23^, ^24^, ^25^, ^26^, ^31^, ^35^, ^36^, ^37^, ^39^, ^42^, ^43^, ^45^, ^50^, ^51^, ^54^, ^55^ A decreasing mortality trend was observed across all time points, favouring home care, but did not reach statistical significance (e.g., 12‐month OR: 0.9; 95% CI: 0.7–1.16; Figure S2).

For readmission, we included five RCTs that provided data at 1 month.^11^, ^14^, ^30^, ^31^, ^51^ Three RCTs at the 2‐month time point,^37^, ^42^, ^43^ nine at 3 months,^13^, ^14^, ^17^, ^20^, ^30^, ^35^, ^39^, ^45^, ^55^ seven at 6 months^11^, ^14^, ^23^, ^26^, ^39^, ^51^, ^56^ and four at 12 months.^18^, ^24^, ^25^, ^35^, ^48^ Overall estimated effects on the risk of readmission suggested a non‐significant decrease in risk measured at several time points or readmission at 1 month (OR 1.08, 95% CI 0.73‐1.58) and 12 months (OR 1.16, 95% CI 0.80‐1.67) (I^2^ at 12 months = 14%). Mortality at 1 month (OR 0.82, 95% CI 0.54‐1.25) and 12 months (OR 0.86, 95% CI 0.6‐1.25), heterogeneity was minimal across time points (I^2^_=_ 0%) (Figure S3).

**FIGURE 3:**
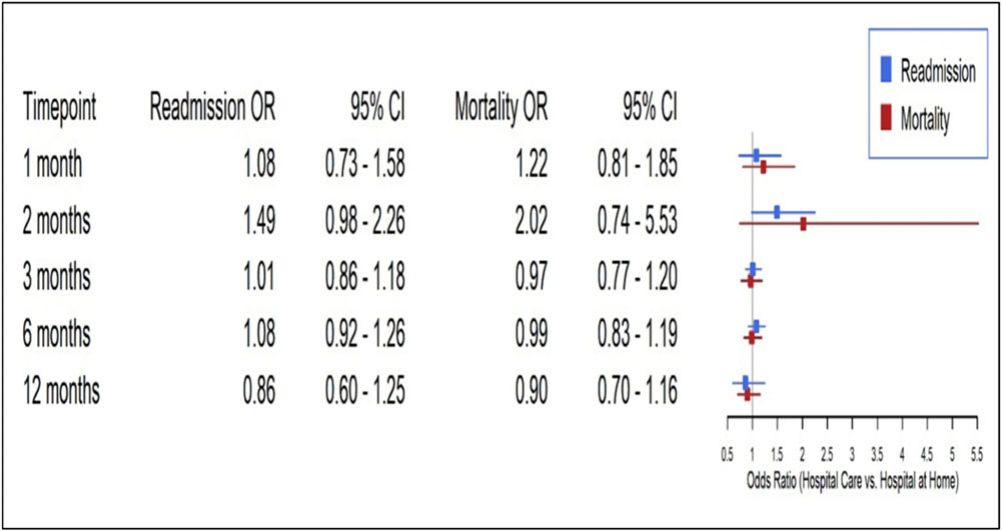
Odds ratios of mortality and readmission across timepoints (X axis: OR).

Thus, home care is non‐inferior to hospital care in terms of mortality and readmission (for selected patients), the results for both outcomes are presented in Figure 3.

### 4.2 Length of stay in care

**FIGURE 4:**
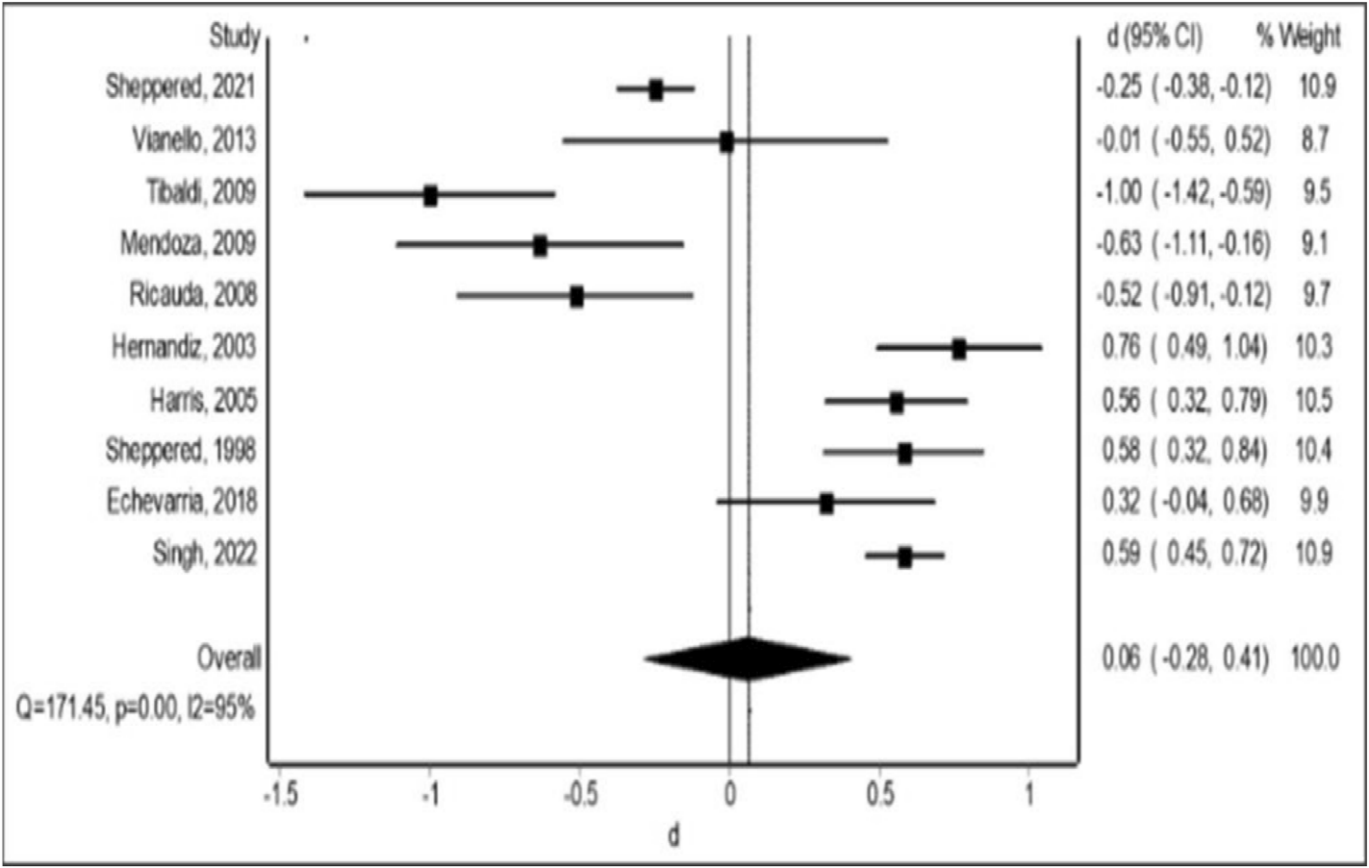
Overall mean difference of length of stay in days in the care.

Our meta‐analysis of 10 RCTs (n = 2547 participants) indicated the mean length of stay was not statistically different compared to HaH with the hospital group (mean difference, 0.06; 95% CI‐0.28‐0.41; I^2^ = 90%; Figure 4).

### 4.3 Emergency attendance at 3 months

Meta‐analysis of emergency attendance was performed using three RCTs (n = 2334 participants).^14^, ^28^, ^37^ The overall effect was inconclusive (2.28 [95% CI: 0.77–6.78] Figure S4) due to the overlapping confidence intervals and crossing the null value, along with high heterogeneity (I^2^ = 90%).

### 4.4 Patient and carer satisfaction

The meta‐analysis for patient satisfaction across different RCTs found no statistical difference along with substantial heterogeneity (I^2^ = 78%) (see Figure S5). However, the HAH consistently showed high patient satisfaction. For example, in an RCT by Ricauda et al. (2008), 94% rated the home care as “very good/excellent,” while 88% rated hospital care as “very good/excellent.”^26^

Carers generally reported high satisfaction with HAH (e.g., 92.7% in Ojoo et al. (2002),^39^ with sustained satisfaction at 3–6 months in Zimmer et al. (1985).^56^ In some comparisons, Carer satisfaction was slightly lower than that of hospital care.^44^ The evidence around caregiver burden was mixed, where Moss et al. (2023) reported no difference between the two groups,^47^ Singh et al. found a decline in total hours of unpaid help (mean difference of −62.76 h, 95% CI − 224.61 to 99.09).^10^ A more detailed description is shown in Table S4.

A qualitative study was conducted following an RCT that compared (HAH) with hospital inpatient treatment.^46^ Thirty‐four elderly patients and 29 carers participated in semi‐structured interviews to examine how older adults and their informal caregivers manage acute health events. Findings indicated that patients and caregivers frequently collaborate with healthcare providers; however, their involvement in comprehensive geriatric assessment (CGA) processes is limited. Caregivers frequently encounter challenges in decision‐making, especially when patients experience confusion. The study suggested that enhancing caregiver involvement could improve healthcare coherence and outcomes.^46^

### 4.5 Cost and cost‐effectiveness

A total of 14 RCTs focused on the economic impact and cost comparison between VWs/HaH.^10^, ^13^, ^17^, ^21^, ^24^, ^25^, ^26^, ^28^, ^37^, ^40^, ^42^, ^44^, ^49^, ^52^ Their findings highlight the various opportunities for cost savings in patient care across healthcare systems in different geographic distributions. Table S5 summarizes potential savings in patient care costs from various RCTs in different countries. Vianello et al (2013) reported significantly lower costs for home care compared to hospital care of respiratory tract infection in patients with neuromuscular disease (NMD) (*p* < 0.001),^17^ and Echevarria (2018) demonstrated that mean 90‐day costs were £1016 lower in HaH, primarily due to reduced hospital bed days: HaH = 1 (IQR 1–7), UC = 5 (IQR 2–12) (*p <* 0.001).^13^ Based on quality‐adjusted life years, the probability of HAH being cost‐effective was 90%.^13^ Singh et al. (2022) found that societal costs were significantly lower (−£3083, 95% CI: −5880 to −287).^10^ Medication costs were factored into some of the economic analyses, like Mendoza et al. (2008),^24^ Skwarska et al. (2000)^42^ and Nicholson et al. (2001).^40^

### 4.6 QoL and QALYs‐pain and physical health

Sixteen RCTs evaluated the health‐related quality of life (HRQL) across various patient groups with a wide range of health conditions (Table S6). The QoL findings were mixed; some RCTs showed less pain,^22^ improved functionality^48^ and social activity.^53^ Shepherd et al. (1998) RCT on hip replacement patients showed a non‐significant difference with slightly higher social activity in home care (82% *vs*. 78%).^53^ The HaH, in particular conditions such as COPD or heart failure,^22^, ^23^ and others found comparable QoL outcomes (no significant differences) between groups.^10^, ^13^, ^25^, ^30^, ^53^ Overall, HaH may enhance the patient experience without compromising clinical outcomes.

Several RCTs have consistently shown that hospital‐at‐home is not inferior to managing COPD exacerbations^13^, ^19^, ^20^, ^45^ People over 65 and those at lesser risk (DECAF score < 4) benefited the most, according to the subgroup studies.^26^ Home care for this condition has been shown to reduce costs by 29–38%.^13^, ^28^

Several RCTs have highlighted the role of pharmaceutical care within HaH programs, although medication safety monitoring was often underreported. Singh et al. (2022) evaluated a multidisciplinary HaH model that included pharmacists, geriatricians and nurses.^10^ Similarly, an intervention for high‐risk patients involved a part‐time pharmacist,^14^ while other trials maintained standard pharmacologic treatments,^17^, ^20^, ^23^ with drug costs factored into economic analyses.^24^, ^40^, ^42^ Despite these efforts, most studies lacked explicit tracking of medication adherence or adverse drug events (ADEs).^35^, ^42^

**FIGURE 5:**
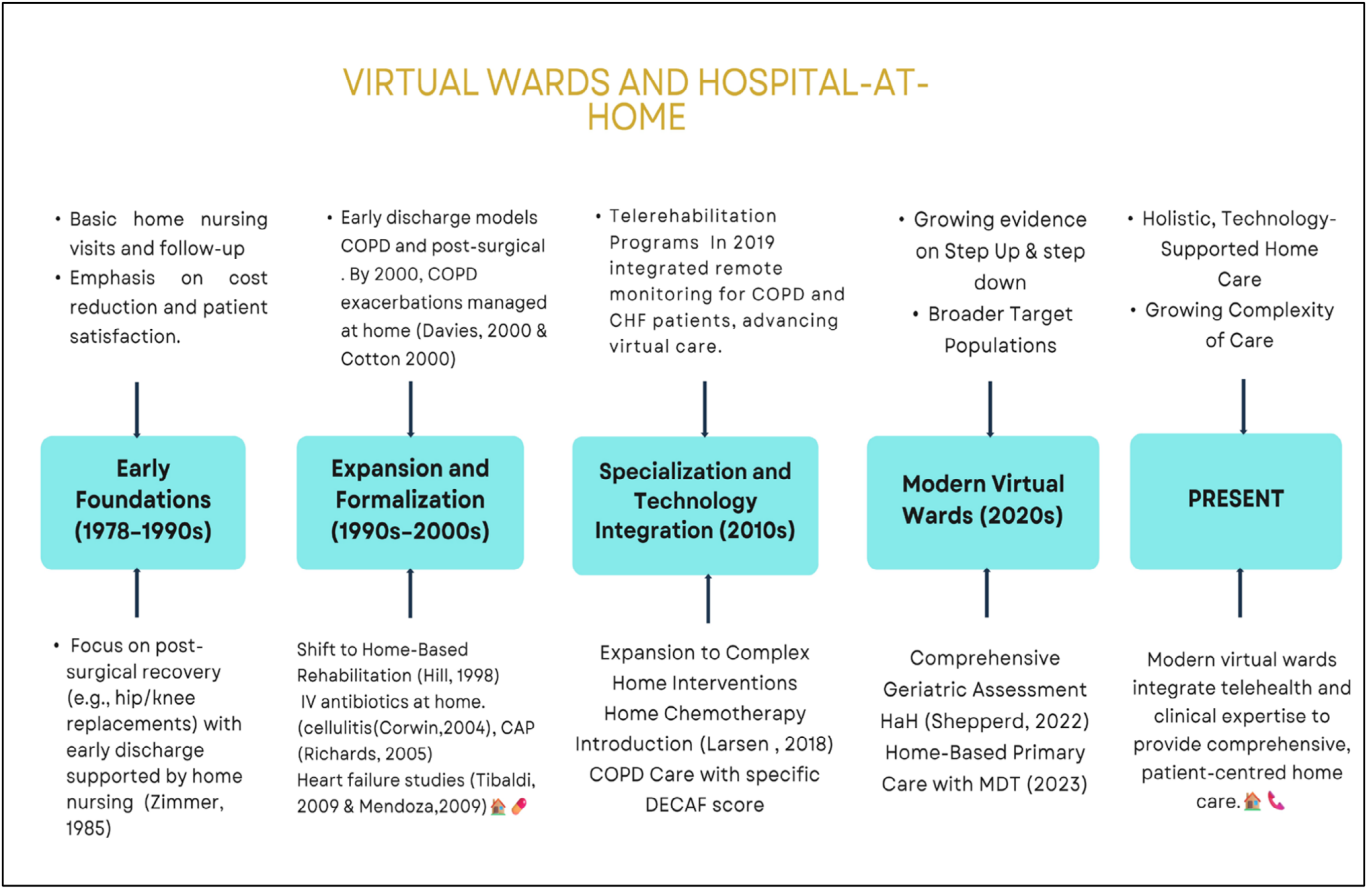
Virtual wards and hospital at home evolution in research.

We also developed a timeline of the evolution of the model of care based on the included RCTs. The more recent advances include more technology integration, particularly telemonitoring.^57^, ^58^, ^59^ However, standardizing the model of interest in the included RCTs (PICO approach) was essential for comparing outcomes and enabling meta‐analysis of measured endpoints (Figure 5).

## 5 DISCUSSION

### 5.1 Summary of key findings

This systematic review and meta‐analysis of 47 RCTs (9749 participants) demonstrated that VWs and HaH models had shown non‐inferiority to hospital care with no statistically significant differences in mortality, readmission, emergency attendance or patient satisfaction evaluated at time points ranging from 1 to 12 months. The narrative synthesis suggests potential benefits of HaH/VW in terms of cost savings, quality of life and patient and carer satisfaction. For caregiver burden, the results were mixed.^15^, ^19^, ^30^, ^32^, ^35^, ^38^, ^41^, ^48^

Consistent with our findings of non‐inferiority to hospital care, Shi and colleagues (2024)^60^ showed a similar conclusion in mortality outcomes. They also found that HaH interventions are probably equivalent to hospital‐based inpatient care in terms of clinical outcomes. Another review by Arsenault‐ Lapierre et al. (2021) reported comparable mortality risks between HaH and traditional hospital settings.^61^ However, this is different from the review by Federman et al. (2018), who suggested that HaH care leads to clinically significant decreases in mortality compared to inpatient care.^62^ Another review by Huntley and colleagues (2017) revealed HaH is linked to fewer emergency department visits and can reduce hospital readmissions and overall healthcare costs.^63^

The findings across RCTs were inconsistent, which highlights the variability in HaH models and patient populations. As noted by Leong and colleagues (2021),^7^ the effectiveness of HaH models can vary based on the type of program implemented (early supported discharge *vs*. admission avoidance). However, the benefits in terms of mortality and readmissions were primarily observed in heart failure patients. Meta‐analysis of 24 RCTs found that VW transitional care significantly decreased hospital readmissions and mortality in heart failure patients.^64^

HaH models have shown effectiveness in managing acute decompensation of chronic heart failure and post‐surgical care, although key limitations should be noted. For example, the RCT by García‐Soleto et al. (2013) was constrained by its small, single‐centred sample,^18^ while Tibaldi et al. (2009), by including healthier elderly patients, might have overestimated benefits,^23^ Mendoza et al. (2009) lacked long‐term follow‐up despite showing short‐term readmission reductions,^24^ and the pilot study was underpowered for clinical outcomes.^34^ These limitations mirror systematic review findings that highlight HaH's potential for reducing hospital stays and costs.^65^, ^66^, ^67^ However, it is essential to emphasize the need for larger, longer‐duration trials with standardized patient selection to address current evidence gaps in real‐world applicability.

For COPD RCTs, three main limitations were observed: exclusion of high‐risk cases (DECAF <= 4 in Echevarria and colleagues (2018),^13^ trial populations that are centred in cities,^20^ and variations in patient selection criteria.^37^ Although patients highly prefer HaH^38^, ^39^, ^68^, ^69^ (partially by factors such as patient education and personalized care plans). Widespread adoption, especially for more severe exacerbations, requires consistent risk‐stratification tools and processes.

Economic evaluations for COPD home care show that the probability of HaH being cost‐effective was 90%^13^, ^28^ although model‐based analyses may overestimate real‐world savings as the trial assumes ideal implementation.^19^ Even though many economic analyses included medication components, they were not broken down into the specific components, limiting the benefits and possibilities of capturing overprescribing issues.^24^, ^40^, ^42^

Several studies have shown that HaH/VW environments increase satisfaction and reduce carer load,^34^, ^38^, ^39^ and patient choice is frequently discussed.^16^, ^38^, ^48^, ^69^ In Echevarria et al. (2018), 90% of patients preferred HaH for future care, and in Ojoo et al. (2002), 91.7% preferred HaH compared to 88.10% for the hospital group. Hospital care had a favourable satisfaction in one RCT,^39^ but HAH often outperforms traditional hospital care in many RCTs.^37^, ^56^

When receiving treatment at home, patients frequently report feeling more psychologically at ease. Stress and anxiety levels are lowered in a familiar setting, especially when providing education to the patient and carer. In Utens et al. (2013),^16^ where the nurse provided counselling and inhaler training, medication compliance and inhalation techniques were addressed, and in Hendricks and Talcott (2011),^21^, ^22^ an educational intervention was provided. However, medication adherence and adverse drug events (ADEs) were not fully reported or measured in many studies that lacked pharmacist input on the team.^30^, ^48^ While virtual care improves satisfaction, the absence of ADE data in the included RCTs highlights a critical gap. Policymakers should mandate ADE monitoring protocols similar to those used in hospital pharmacovigilance.

One additional finding of note is that although satisfaction is high, in one RCT over 25% of patients needed readmission due to poor home care.^17^ Since current healthcare policy emphasizes patient‐centred therapy, these findings are important because effective usage requires strong risk‐stratification approaches to decrease adverse events.

Different cost perspectives have been utilized in studying the cost and cost‐effectiveness of virtual healthcare models. The review's findings on cost savings support existing literature that emphasizes the potential economic advantages of virtual healthcare. Research by Jones et al. (1999)^49^ showed that HaH programs could lead to substantial reductions in healthcare costs, particularly in terms of daily patient care expenses.^49^, ^50^ Some studies considered the cost from the healthcare provider's perspective,^34^, ^52^ while others analysed direct and indirect costs, including health and social care costs, societal costs (which included informal care) and total costs associated with each intervention group.^10^, ^40^ To address this, we standardized the cost to pounds sterling at the time of writing this article, and by focusing on overall cost reductions, we compared VWs and the hospital.

Most included RCTs focused on cities and were conducted in high‐income countries (e.g. the USA, UK & Netherlands)^11^, ^12^, ^20^ raising concerns about scalability in low‐income countries. Recent research in some countries, like Turkey, focused on hospital‐to‐home transition interventions in terms of family and patient education, or discharge planning to reduce readmission rates compared to usual post‐discharge care (instead of hospital care).^70^, ^71^

High heterogeneity was noted in the results of the analyses of patient/carer satisfaction, LoS, and emergency attendance at 3 months. This can be attributed to the unblinded nature of the intervention, and the use of different measurement tools and timing of assessment, resulting in heterogeneity in patient satisfaction (I^2^ = 78%). However, we standardized the LOS definition as days in the allocated care model, and most trials used admission‐avoidance pathways; substantial heterogeneity remained (I^2^ = 90%) for LOS results. This likely reflects variation in patient populations (e.g., single‐condition COPD *vs*. mixed acute illness), methodological factors such as small sample sizes, the skewed nature of length‐of‐stay data, conversions from medians to means and variations in discharge procedures across settings could be the leading causes of heterogeneity. In readmission outcome results, low heterogeneity was observed, which most likely reflects variations in follow‐up duration, case mix and baseline patient risk among studies. It can be seen clearly that the longer the duration of the follow‐up the less the heterogeneity. By contrast, mortality outcomes were consistent across trials, with minimal heterogeneity, reflecting the relative objectivity and uniformity of this outcome measure.

### 5.2 Strengths and limitations of the review

This systematic review and meta‐analysis provide a thorough overview and data synthesis of RCTs, following PRISMA guidelines and utilizing robust statistical methods. The study encompasses a wide range of healthcare settings, thereby enhancing its generalizability. Additionally, the inclusion of around 9749 participants (patients and carers) in the RCT design of studies with no restrictions on geographical destination or language, and the inclusion of early RCTs investigating the effectiveness of VWs, adds weight to the evidence. Standardizing the model of care being studied enhanced the conclusiveness, as many reviews attempted to include more interventions, which led to difficulties in drawing conclusive results. However, differences in how outcomes were measured made it challenging to combine all the data in a meta‐analysis. To address this, a random effect model was utilized in the analysis to account for heterogeneity. However, high heterogeneity was observed for several outcomes. Differences in study populations (e.g., COPD, heart failure, mixed acute illness), the context of the healthcare system in different countries and the intensity of hospital‐at‐home or virtual ward models (ranging from daily in‐person visits to predominantly remote monitoring), and the team characteristics, are likely other causes of variability.

### 5.3 Policy & Research Implications

The findings of this review have several important implications for both clinical practice and policy. The results suggest that virtual wards do not increase mortality rates compared to inpatient hospital care, showing their non‐inferior safety and that virtual wards could be a viable alternative. Current evidence is too inconsistent to make condition‐specific recommendations for the specific patient groups. To guarantee scalability, policymakers should prioritize context‐specific cost‐effectiveness studies that incorporate a more detailed analysis of pharmaceutical components in cost evaluations. In low‐ and middle‐income countries where digital literacy and infrastructure may hinder the delivery of these services, studies should focus on patient and caregiver support mechanisms.

Future RCTs should focus on large‐scale, multi‐centre RCTs with standardized virtual ward models. Additionally, it is crucial to focus on long‐term patient outcomes that extend beyond 12 months. Additionally, future studies should assess the impact of virtual wards on healthcare workforce efficiency and burnout, and the role of pharmaceutical care within the VWs/HaH programs.^6^, ^72^

## 6 CONCLUSION

Overall, this systematic review and meta‐analysis suggest that virtual wards are a safe alternative to inpatient care, as they do not significantly affect or differ in mortality, readmission rates, emergency visits or length of stay compared to hospital care. These findings offer strong support for the cautious and strategic expansion of virtual healthcare models. However, the successful implementation of virtual wards requires the development of clear regulatory frameworks to ensure patient safety and accountability.

## AUTHOR CONTRIBUTIONS

Rana A. Malhis, Stuart E. Bond, and Mamoon A. Aldeyab developed the study concept and review protocol. Rana A. Malhis and Mamoon A. Aldeyab developed the search strategy. Rana A. Malhis, Ahmed A. Sadeq and Rani Shatnawi searched the databases, selected studies, and extracted data. Rana A. Malhis and Syed Shahzad Hasan analysed the data. All authors contributed significantly to the interpretation of the data. Rana A. Malhis drafted the manuscript. Stuart E. Bond, Ahmed A. Sadeq, Rani Shatnawi, Barbara R. Conway, Syed Shahzad Hasan, Mamoon A. Aldeyab contributed to the revision of the manuscript for important intellectual content. Mamoon A. Aldeyab, Stuart E. Bond and Syed Shahzad Hasan supervised the work. All authors agreed and approved the final version of the article.

## CONFLICT OF INTEREST STATEMENT

The authors have no relevant affiliations or financial involvement with any organization or entity with a financial interest in or financial conflict with the subject matter or materials discussed in the manuscript. This includes employment, consultancies, honoraria, stock ownership or options, expert testimony, grants or patents received or pending or royalties.

## Supporting information


**Table S1.** PICO approach: Population, Intervention, Comparator, and Outcome inclusion and exclusion criteria.
**Table S2.** Search strategy for databases.
**Table S3.** Reason for exclusion at full‐text screening stage (sample articles).
**Table S4.** Patient and Caregiver Satisfaction in Hospital at Home (HaH) *vs.* Hospital.
**Table S5.** Summary of Costs and Cost Savings of Hospital at Home/Virtual Ward (HaH/VW) Compared to Hospital Care.
**Table S6.** Summary of Quality of Life (QoL) Findings for Home Care Compared to Hospital Care.
**Figure S1.** Publication Bias Assessment of Readmission at 3 Months Funnel Plot.
**Figure S2.** Pooled Odds Ratios of Mortality at 1, 2, 3, 6, and 12 months.
**Figure S3.** Pooled Odds Ratios of Readmission at 1, 2, 3, 6, and 12 months.
**Figure S4.** Forest plot of emergency attendance at 3 months across different studies comparing hospital care to home care.
**Figure S5.** Forest plot of patient satisfaction at the end of treatment.

## Data Availability

The data that supports the findings of this study are available in the supplementary material of this article
