# Supplementary material for: Virtual healthcare compared to hospital care for acute and post‐acute illness in adults: A systematic review and meta‐analysis of randomized controlled trials
Source: Br J Clin Pharmacol. 2025 Nov 29;92(2):374–95. doi: 10.1002/bcp.70348 (PMC12850592; doi:10.1002/bcp.70348)
Supplement: Supplementary file 1 — Table S1. PICO approach: Population, Intervention, Comparator, and Outcome inclusion and exclusion criteria. Table S2. Search strategy for databases. Table S3. Reason for exclusion at full‐text screening stage (sample articles). Table S4. Patient and Caregiver Satisfaction in Hospital at Home (HaH) vs. Hospital. Table S5. Summary of Costs and Cost Savings of Hospital at Home/Virtual Ward (HaH/VW) Compared to Hospital Care. Table S6. Summary of Quality of Life (QoL) Findings for Home Care Compared to Hospital Care. Figure S1. Publication Bias Assessment of Readmission at 3 Months Funnel Plot. Figure S2. Pooled Odds Ratios of Mortality at 1, 2, 3, 6, and 12 months. Figure S3. Pooled Odds Ratios of Readmission at 1, 2, 3, 6, and 12 months. Figure S4. Forest plot of emergency attendance at 3 months across different studies comparing hospital care to home care. Figure S5. Forest plot of patient satisfaction at the end of treatment. [file BCP-92-374-s001.docx]

**List of supplementary tables**

| **Table No.** | **Content Description-supplementary tables** |
| --- | --- |
| **Table S1** | PICO approach: Population, Intervention, Comparator, and Outcome inclusion and exclusion criteria. |
| **Table S2** | Search strategy for databases. |
| **Table S3** | Reason for exclusion at full-text screening stage (sample articles). |
| **Table S4** | Patient and Caregiver Satisfaction in Hospital at Home (HaH) vs. Hospital. |
| **Table S5** | Summary of Costs and Cost Savings of Hospital at Home/Virtual Ward (HaH/VW) Compared to Hospital Care. |
| **Table S6** | Summary of Quality of Life (QoL) Findings for Home Care Compared to Hospital Care. |

**Table S1. PICO approach: Population, intervention, and comparator inclusion and exclusion criteria.**

| **Parameter** | **Population** | **Intervention** | **Comparator** | **Outcome** |
| --- | --- | --- | --- | --- |
| Inclusion criteria | Any males and females aged >=18 years old admitted to the virtual ward are going to be included. | Admission to the virtual ward/hospital at home from community healthcare teams (admission avoidance/step-up model) or post-hospital discharge (early discharge/step-down mode) for the management of acute or post-acute illness where otherwise patient should be in the hospital. | Hospital inpatient care. | Primary: Readmission, Mortality, emergency attendance and length of stay  Secondary: quality of life, patient and carer satisfaction, and costs. |
| Exclusion criteria | Paediatric patients (<18 years old). pregnant women. palliative end-of-life care. mental illness patients.  people with long-term care needs unless they require admission to the hospital for an acute episode of care | Studies that studied remote monitoring without at least one home visit by health care staff.  Studies that did not have at least a single home-based intervention   Services providing long-term care.  Self-care by the patient in their home (e.g., self-administration of medications) |  |  |

**Table S2. Search strategy for databases.**

| **Database** | **Search strategy** | **Final search on 15 October 2024** |
| --- | --- | --- |
| **Pubmed** | ("Home Care Services, Hospital-Based"[Mesh] OR "Home Health Nursing"[Mesh] OR "Home Care Services"[Mesh] OR "virtual ward*"[tiab] OR “community ward*”[tiab] OR "hospital at home"[tiab] OR hospital-at-home[tiab] OR "community-based care"[tiab] OR “community ward”[tiab] OR “community care” OR “transitional care”[tiab] OR “care transition*”[tiab] OR homecare[tiab] OR "Community Health Services"[Mesh] OR "Professional-Patient Relations"[Mesh])  AND  ("Patient Discharge"[Mesh] OR "Hospitalization"[Mesh] OR “discharge planning”[tiab] OR “admission avoidance”[tiab] OR “readmission avoidance”[tiab] OR “prevent readmission*”[tiab] OR “early discharge”[tiab] OR “patient admission*”[tiab] OR discharge*[tiab] OR admission*[tiab])  AND  (Randomized controlled trial[pt] OR controlled clinical trial[pt] OR randomized[tiab] OR randomised[tiab] OR placebo[tiab] OR "drug therapy"[sh] OR "therapeutic use"[sh] OR randomly[tiab] OR trial[tiab] OR groups[tiab])  NOT  (Animals[Mesh] not (Animals[Mesh] and Humans[Mesh])) | 7,626  results |
| **CINAHL** | ("Home Care Services, Hospital-Based"[Mesh] OR "Home Health Nursing"[Mesh] OR "Home Care Services"[Mesh] OR "virtual ward* “ OR "community ward* “ OR "hospital at home “ OR hospital-at-home OR "community-based care “ OR "community ward “ OR "community care" OR "transitional care “ OR "care transition* “ OR homecare OR "Community Health Services"[Mesh] OR "Professional Patient Relations"[Mesh])  AND  ("Patient Discharge"[Mesh] OR "Hospitalization"[Mesh] OR "discharge planning “ OR "admission avoidance “ OR "readmission avoidance “ OR "prevent readmission* “ OR "early discharge “ OR "patient admission* “ OR discharge* OR admission*)  AND   (Randomized controlled trial[pt] OR controlled clinical trial[pt] OR randomized OR randomised OR placebo OR "drug therapy"[sh] OR "therapeutic use"[sh] OR randomly OR trial OR groups)   NOT  (Animals[Mesh] not (Animals[Mesh] and Humans[Mesh])) | 1,333 results |
| **CENTRAL** | "Home Care Services, Hospital-Based" OR "Home Health Nursing" OR "Home Care Services" OR "virtual ward" OR "community ward" OR "hospital at home" OR  "community-based care" OR "community ward" OR "community care" OR "transitional care" OR "care transition" OR homecare OR "Community Health Services" OR "Professional Patient Relations"  "Home Care Services, Hospital-Based" OR "Home Health Nursing" OR "Home Care Services" OR "virtual ward" OR "community ward" OR "hospital at home" OR  "community-based care" OR "community ward" OR "community care" OR "transitional care" OR "care transition" OR homecare OR "Community Health Services" OR "Professional Patient Relations"   AND   ("Patient Discharge" OR "Hospitalization" OR "discharge planning" OR "admission avoidance" OR "readmission avoidance" OR "prevent readmission" OR "early discharge" OR "patient admission" OR discharge OR admission)  AND  (Randomized controlled trial OR controlled clinical trial OR randomized OR randomised OR placebo OR "drug therapy" OR "therapeutic use" OR randomly OR trial OR groups) | 1,817 trials |
| **EMBASE** | ('Home Care Services, Hospital-Based'/exp OR 'Home Health Nursing'/exp OR 'Home Care Services'/exp OR 'virtual ward*':ti,ab OR 'community ward*':ti,ab OR 'hospital at home':ti,ab OR hospital-at-home:ti,ab OR 'community-based care':ti,ab OR 'community ward':ti,ab OR  'Community care'   OR 'transitional care':ti,ab OR 'care transition*':ti,ab OR homecare:ti,ab OR 'Community Health Services'/exp OR 'Professional-Patient Relations'/exp)  AND  ('Patient Discharge'/exp OR Hospitalization/exp OR 'discharge planning':ti,ab OR 'admission avoidance':ti,ab OR 'readmission avoidance':ti,ab OR 'prevent readmission*':ti,ab OR 'early discharge':ti,ab OR 'patient admission*':ti,ab OR discharge*:ti,ab OR admission*:ti,ab)  AND  (term:it OR term:it OR randomized:ti,ab OR randomised:ti,ab OR placebo:ti,ab OR "Drug Therapy" OR "Therapeutic Use" OR randomly:ti,ab OR trial:ti,ab OR groups:ti,ab)  NOT  (Animals/exp NOT (Animals/exp AND Humans/exp)) | 11,465 results |

**Table S3. Reason for exclusion at full text screening stage (sample articles).**

| **Author, year** | **Title** | **Reason** | **Reference** |
| --- | --- | --- | --- |
| **Yadav et al., 2023** | The impact of a hybrid hospital at home program in reducing subacute rehabilitation referrals | Not randomised - patient already in VW compared to hospital inpatients | Yadav RR, Mahyoub MA, Capriotti MW, Berio-Dorta RL, Dougherty K, Shukla A. The impact of a hybrid hospital at home program in reducing subacute rehabilitation referrals. medRxiv. 2023 Jun 3:2023-05. |
| **Toles et al., 2023** | Connect-Home transitional care from skilled nursing facilities to home: a stepped wedge, cluster randomized trial | Comparator is the discharged patients (not hospital based) | Toles M, Preisser JS, Colón‐Emeric C, Naylor MD, Weinberger M, Zhang Y, Hanson LC. Connect‐Home transitional care from skilled nursing facilities to home: A stepped wedge, cluster randomized trial. Journal of the American Geriatrics Society. 2023 Apr;71(4):1068-80. |
| **Elena Ramírez-Maldonado et al., 2022** | Home care/outpatient versus hospital admission in mild acute pancreatitis: protocol of a multicentre, randomised  controlled trial (PADI_2 trial) | Protocol for a study, no results | Ramírez-Maldonado E, Rodrigo-Rodrigo M, Gordo SL, Sanchez A, Llanos DC, Sanchez R, Vaz J, Fondevila C, Jorba-Martin R. Home care/outpatient versus hospital admission in mild acute pancreatitis: protocol of a multicentre, randomised controlled trial (PADI_2 trial). BMJ open. 2023 Jun 1;13(6):e071265 |
| **J. Helberg et al., 2023** | Heart failure management at home: a non-randomised prospective case-controlled trial (HeMan at Home) | Not randomised | Helberg J, Bensimhon D, Katsadouros V, Schmerge M, Smith H, Peck K, Williams K, Winfrey W, Nanavati A, Knapp J, Schmidt M. Heart failure management at home: a non-randomised prospective case–controlled trial (HeMan at Home). Open Heart. 2023 Dec 1;10(2):e002371. |
| **X. Yao et al., ,2022** | Effect of hospital-at-home vs. traditional brick-and-mortar hospital care in acutely ill adults: study protocol for a pragmatic randomized controlled trial | Study protocol -no results | Yao X, Paulson M, Maniaci MJ, Dunn AN, Nelson CR, Behnken EM, Hart MS, Sangaralingham LR, Inselman SA, Lampman MA, Dunlay SM. Effect of hospital-at-home vs. traditional brick-and-mortar hospital care in acutely ill adults: study protocol for a pragmatic randomized controlled trial. Trials. 2022 Jun 16;23(1):503. |
| **H. Beer et al., 2021** | The development of a home-based therapeutic platform for multiple myeloma | Descriptive of H@H | Beer H, Routledge D, Joyce T, Furphy EJ, Combe N, Ritchie D, Khot A, Lim SM, Montalto M, Harrison SJ. The development of a home-based therapeutic platform for multiple myeloma. Expert Review of Hematology. 2021 Dec 2;14(12):1129-35. |
| **M. Auaiset al., 2020** | The stronger at home study: A feasibility randomized controlled trial of homebased physiotherapy program for patients after hip fractures | Poster abstract-embase ( not enough results data) | Auais M, Miller J, Varette K, Zaman MS. THE STRONGER AT HOME STUDY: A FEASIBILITY RANDOMIZED CONTROLLED TRIAL OF HOMEBASED PHYSIOTHERAPY PROGRAM FOR PATIENTS AFTER HIP FRACTURES. InOSTEOPOROSIS INTERNATIONAL 2020 Dec 1 (Vol. 31, No. SUPPL 1, pp. S571-S571). 236 GRAYS INN RD, 6TH FLOOR, LONDON WC1X 8HL, ENGLAND: SPRINGER LONDON LTD. |
| **C. B. Mogensen et al., 2018** | Admission rates in a general practitioner-based versus a hospital specialist based, hospital-at-home model: ACCESS, an open-labelled randomised clinical trial of effectiveness | The comparison between two H@H (GP Vs Hospital) | Mogensen CB, Ankersen ES, Lindberg MJ, Hansen SL, Solgaard J, Therkildsen P, Skjøt-Arkil H. Admission rates in a general practitioner-based versus a hospital specialist based, hospital-at-home model: ACCESS, an open-labelled randomised clinical trial of effectiveness. Scandinavian journal of trauma, resuscitation and emergency medicine. 2018 Apr 5;26(1):26. |
| **A. Escartín et al., 2018** | Home hospitalization for the surgical and conservative treatment of acute calculous cholecystitis | Retrospective analysis | Escartín A, Mías MC, González M, Cuello E, Pinillos A, Muriel P, Mestres N, Villalobos R, Olsina JJ. Home hospitalization for the surgical and conservative treatment of acute calculous cholecystitis. Surgical Practice. 2018 May;22(2):52-9. |
| **Federman et al., 2018** | Bundled Hospital-at-Home and Transitional Care Program Is Associated with Reduced Rate of Hospital Readmission | Cohort (matching controls) | Federman AD, Soones T, DeCherrie LV. Bundled Hospital-at-Home and Transitional Care Program Is Associated with Reduced Rate of Hospital Readmission. JAMA. 2018;178:1033-40. |
| **S. Shepperd et al., 2017** | A multi-centre randomised trial to compare the effectiveness of geriatrician-led admission avoidance hospital at home versus inpatient admission | Study protocol -no results | Shepperd S, Cradduck-Bamford A, Butler C, Ellis G, Godfrey M, Gray A, Hemsley A, Khanna P, Langhorne P, McCaffrey P, Mirza L. A multi-centre randomised trial to compare the effectiveness of geriatrician-led admission avoidance hospital at home versus inpatient admission. Trials. 2017 Oct 23;18(1):491. |
| **C. Escobar et al., 2017** | 30-day ED visit and readmission rate after a hospital at home admission vs traditional hospitalization | Post -discharge  comparison | Escobar C, Soones TN, Leff B, Marshall J, Silversmith G, Siu AL, Wajnberg A, Decherrie L. 30-day Ed Visitand Readmission Rate After A Hospital At Home Admission Vs Traditional Hospitalization. Injournal OF General INTERNAL MEDICINE 2017 Apr 1 (Vol. 32, Pp. S726-s726). 233 Spring St, New York, Ny 10013 Usa: Springer. |
| **Actrn 2020** | Evaluating the Further Enabling Care at Home (FECH) post-discharge program as a way to support carers of older hospital patients | A register of trial- no results  post‐discharge telephone caregiving support program | Hill AM, Moorin R, Slatyer S, Bryant C, Hill K, Waldron N, Aoun S, Kamdar A, Grealish L, Reberger C, Jones C, Bronson M, Bulsara MK, Maher S, Claverie T, Moyle W. Evaluating the provision of Further Enabling Care at Home (FECH+) for informal caregivers of older adults discharged home from hospital: protocol for a multicentre randomised controlled trial. BMJ Open. 2021 Jun 21;11(6):e046600; PMCID: PMC8217916. |
| **P. Bernocchi et al., 2016** | A multidisciplinary telehealth program in patients with combined chronic obstructive pulmonary disease and chronic heart failure: Study protocol for a randomized controlled trial | Study protocol | Bernocchi, P., Scalvini, S., Galli, T. *et al.* A multidisciplinary telehealth program in patients with combined chronic obstructive pulmonary disease and chronic heart failure: study protocol for a randomized controlled trial. *Trials* **17**, 462 (2016) |
| **M. J. Carrington et al., 2013** | A multicenter, randomized trial of a nurse-led, home-based intervention for optimal secondary cardiac prevention suggests some benefits for men but not for women: the Young at Heart study | Usual post -discharge care | Carrington MJ, Chan YK, Calderone A, Scuffham PA, Esterman A, Goldstein S, Stewart S; Young at Heart Investigators. A multicenter, randomized trial of a nurse-led, home-based intervention for optimal secondary cardiac prevention suggests some benefits for men but not for women: the Young at Heart study. Circ Cardiovasc Qual Outcomes. 2013 Jul;6(4):379-89. doi: 10.1161/CIRCOUTCOMES.111.000006. Epub 2013 Jul 2. PMID: 23819955. |
| **Y. K. Chan et al., 2012** | Exploring the potential to remain "Young @ Heart": initial findings of a multi-centre, randomised study of nurse-led, home-based intervention in a hybrid health care system | The control group in the Young @ Heart study received usual care (UC) in the community. This care was provided by the patients' general physicians and other healthcare team members without the explicit involvement of a cardiac nurse | Chan YK, Stewart S, Calderone A, et al. Exploring the potential to remain "Young @ Heart": initial findings of a multi-centre, randomised study of nurse-led, home-based intervention in a hybrid health care system. *Int J Cardiol*. 2012;154(1):52-58. |
| **C. M. Utens et al., 2010** | Effectiveness and cost-effectiveness of early assisted discharge for chronic obstructive pulmonary disease exacerbations: the design of a randomised controlled trial | Study protocol- design | Utens CM, Goossens LM, Smeenk FW, et al. Effectiveness and cost-effectiveness of early assisted discharge for chronic obstructive pulmonary disease exacerbations: the design of a randomised controlled trial. *BMC Public Health*. 2010;10:618 |
| **K. Allen et al., 2009** | A randomized trial testing the superiority of a postdischarge care management model for stroke survivors | The control group is discharged from hospital | Allen K, Hazelett S, Jarjoura D, et al. A randomized trial testing the superiority of a postdischarge care management model for stroke survivors. *J Stroke Cerebrovasc Dis*. 2009;18(6):443-452. |
| **S. Barnason et al., 2006** | Impact of a telehealth intervention to augment home health care on functional and recovery outcomes of elderly patients undergoing coronary artery bypass grafting | Both HCI and control subjects received HHC | Barnason S, Zimmerman L, Nieveen J, Hertzog M. Impact of a telehealth intervention to augment home health care on functional and recovery outcomes of elderly patients undergoing coronary artery bypass grafting. *Heart Lung*. 2006;35(4):225-233. |
| **Escartín et al., 2018** | Home hospitalization for the surgical and conservative treatment of acute calculous cholecystitis | Not RCT | Escartín, A., Mías, M.-C., González, M., Cuello, E., Pinillos, A., Muriel, P., Mestres, N., Villalobos, R. and Olsina, J.-J. (2018), Home hospitalization for the surgical and conservative treatment of acute calculous cholecystitis. Surg Pract, 22: 52-59. |
| **Donald et al., 1995** | Hospital at-home: a randomized controlled trial. | Compared to usual discharge | Donald IP, Baldwin RN, Bannerjee M. Gloucester hospital-at-home: a randomized controlled trial. *Age Ageing*. 1995;24(5):434-439. |
| **Åsa Karlsson PT et al.,**  **2016** | Effects of Geriatric Interdisciplinary Home Rehabilitation on Walking Ability and Length of Hospital Stay After Hip Fracture: A Randomized Controlled Trial | The control group included outpatiet care when needed | Karlsson Å, Berggren M, Gustafson Y, Olofsson B, Lindelöf N, Stenvall M. Effects of geriatric interdisciplinary home rehabilitation on walking ability and length of hospital stay after hip fracture: a randomized controlled trial. Journal of the American Medical Directors Association. 2016 May 1;17(5):464-e9. |
| **Yih-Kai Chan et al. 2012** | randomised study of nurse-led, home-based intervention in a hybrid health care system | * the control is not inpatient | Chan YK, Stewart S, Calderone A, Scuffham P, Goldstein S, Carrington MJ, Young@ Heart Investigators. Exploring the potential to remain “Young@ Heart”: Initial findings of a multi-centre, randomised study of nurse-led, home-based intervention in a hybrid health care system. International journal of cardiology. 2012 Jan 12;154(1):52-8. |
| **Mie Nordly et al. 2019** | *Systematic fast-track transition from oncological  treatment to dyadic specialized palliative home care: DOMUS – a randomized clinical trial * | the control is not inpatient | Nordly M, Skov Benthien K, Vadstrup ES, Kurita GP, von Heymann-Horan AB, von der Maase H, Johansen C, Timm H, Kjellberg J, Sjøgren P. Systematic fast-track transition from oncological treatment to dyadic specialized palliative home care: DOMUS–a randomized clinical trial. Palliative medicine. 2019 Feb;33(2):135-49. |
| **Carlos Brotons et al., 2009** | Randomised Clinical Trial of the Effectiveness of a Home-Based Intervention in Patients With Heart Failure: The IC-DOM Study | the control is not inpatient | Brotons C, Falces C, Alegre J, Ballarín E, Casanovas J, Catà T, Martínez M, Moral I, Ortiz J, Pérez E, Rayó E. Randomized clinical trial of the effectiveness of a home-based intervention in patients with heart failure: the IC-DOM study. Revista Española de Cardiología (English Edition). 2009 Apr 1;62(4):400-8. |
| **Bryant-Lukosius et al.,**  **2015** | The clinical effectiveness and cost-effectiveness of clinical nurse specialist-led hospital to home transitional care: a systematic review | Not RCT | Bryant‐Lukosius D, Carter N, Reid K, Donald F, Martin‐Misener R, Kilpatrick K, Harbman P, Kaasalainen S, Marshall D, Charbonneau‐Smith R, DiCenso A. The clinical effectiveness and cost‐effectiveness of clinical nurse specialist‐led hospital to home transitional care: A systematic review. Journal of evaluation in clinical practice. 2015 Oct;21(5):763-81. |
| **Levine et al., 2022** | Remote vs In-home Physician Visits for Hospital-Level Care at Home A Randomized Clinical Trial | Both arms are Care at home based interventions | Levine DM, Paz M, Burke K, Beaumont R, Boxer RB, Morris CA, Britton KA, Orav EJ, Schnipper JL. Remote vs in-home physician visits for hospital-level care at home: a randomized clinical trial. JAMA Network Open. 2022 Aug 1;5(8):e2229067-. |
| **Ayden et al., 2013** | The relationship between care burden and social support in Turkish Alzheimer patients family caregivers: Cross-sectional study | Cross-sectional study | Yurtsever S, Özge A, Kara A, Yandim A, Kalav S, Yesil P. The relationship between care burden and social support in Turkish Alzheimer patients family caregivers: Cross-sectional study. Journal of Nursing Education and Practice. 2013 Sep 1;3(9):1. |

#### **Table S4. Patient and Caregiver Satisfaction in Hospital at Home (HaH) vs. Hospital**

| **Study ID** | **Outcome Measure** | **HaH** | **Hospital** |
| --- | --- | --- | --- |
| **Echevarria et al., 2018** | Patient preference | 90% preferred HaH for future care. | – |
| **Board et al., 2000** | Patient satisfaction | Significantly higher. | Lower compared to HaH. |
|  | Carer satisfaction | Significantly higher. | Lower compared to HaH. |
| **Sue Palmer Hill et al., 2000** | Patient satisfaction | Higher satisfaction, less family burden. | Lower satisfaction. |
| **Ojoo et al., 2002** | Patient satisfaction | 91.70% | 88.10% |
|  | Carer satisfaction | 92.70% | 91.30% |
| **Skwarska et al., 2000** | Patient satisfaction | 69% "completely satisfied"; 90% felt care was better at home. | – |
| **Hernandiz et al., 2003** | Patient satisfaction | Higher satisfaction. | Lower satisfaction. |
| **Ricauda et al., 2008** | Satisfaction at discharge | 94% rated "very good/excellent." | 88% rated "very good/excellent." |
| **Zimmer et al., 1985** | Patient satisfaction | Higher at 6 months. | Lower compared to HaH. |
|  | Caretaker satisfaction | Significantly higher at 3 and 6 months. | Lower compared to HaH. |

**Table S5. Summary of Costs and Cost Savings of Hospital at Home/Virtual Ward (HaH/VW) Compared to Hospital Care**

| **Study ID** | **Country** | **Total Cost (Hospital)** | **Total Cost (VW/HaH)** | **Cost Saving** | **Daily Cost (Hospital)** | **Daily Cost (VW/HaH)** |
| --- | --- | --- | --- | --- | --- | --- |
| **Singh et al., 2022** | **UK** | – | – | -£2,265 | £455 | £317 |
| **Vianello et al., 2013** | **Italy** | €8,890 (±€10,992.7) | €542 (±€258.5) | -€8,348 | €1,060 (±€592.5) | €65.3 (±€18.6) |
| **Hendricks et al., 2011** | **US** | $10,143 | $7,830 | -$2,313 | – | – |
| **Board et al., 2000** | **Australia** | $3,614 | $1,764 | -$1,850 | – | – |
| **Jones et al., 1999a** | **UK** | £2,557 | £3,660 | -£1,071 | £134 (£216) | £207 (£334) |
| **Skwarska et al., 2000** | **England** | £1,753 | £877 | -£876 | – | – |
| **Bonnema et al., 1998** | **Netherlands** | $4,382 | $3,062 | -$1,320 | – | – |
| **Mendoza et al., 2009** | **Spain** | €4,502 (±€2,153) | €2,541 (±€1,334) | -€1,961 | – | – |
| **Patel et al., 2008** | **Sweden** | Median €5,110 | Median €1,122 | -€3,988 | – | – |
| **Puig-Junoy et al., 2007** | **Spain** | €1,964 | €1,154 | -€810 | – | – |
| **Aimonino Ricauda 2008** | **Italy** | $1,390.90 | $1,175.90 | -$215 | $151.7 (£125) | $101.4 (£83) |
| **Hernandez et al., 2003** | **Spain** | €2,033 | €1,255 | -€778 | – | – |
| **Nicholson et al.** | **Australia** | – | – | -Aus$1,696 | – | – |

*Note: Values in parentheses represent standard deviations or ranges where applicable. Currency conversions (e.g., £, €, $) are provided as reported in the original studies.*

**Table S6: Summary of Quality of Life (QoL) Findings for Home Care Compared to Hospital Care**

| **Author** | **Test** | **Finding** |
| --- | --- | --- |
| **Skwarska et al., 2000** | Chronic Respiratory Questionnaire (CRQ) | No significant differences between home support and hospital groups at 8-week follow-up. |
| **Tibaldi et al., 2009** | Nottingham Health Profile | HaH: 0.08 (1.36); Hospital: 0.18 (1.94); *P* < 0.046. |
| **Shepperd et al., 2021** | QALYs, EQ-5D-5L | Small non-significant difference. |
| **Shepperd et al., 2021** | EQ-5D-5L VAS | HaH: 56.8 (SD 21.4); Hospital: 55.6 (SD 22.9); mean difference 0.32 (95% CI: -3.08 to 3.73). |
| **Echevarria et al., 2018** | QALYs, EQ-5D-5L | Small non-significant difference favouring HaH. |
| **Talcott et al., 2011** | QoL Changes | HaH: Pain decreased, emotional function improved; Hospital: Pain increased, emotional function declined. |
| **Utens et al., 2012** | EQ-5D at T+4 Days | HaH: 0.664 (SD 0.26); Hospital: 0.713 (SD 0.22). |
| **Hendricks et al., 2011** | Patient-Reported QoL | Similar between home and hospital care. |
| **H. Mendoza et al., 2009** | SF-36 Physical | HaH: 91.4 ± 5.2; Hospital: 93.2 ± 4.6; no significant difference. |
| **Patel et al., 2008** | HRQL | No significant difference. |
| **Singh et al., 2022** | QALYs | Small non-significant difference. |
| **C. Hernandez et al., 2003** | Health-Related QoL | Improved SGRQ and SF-12 scores in home care. |
| **L. Davies et al., 2000** | St George’s Questionnaire | Higher scores in home care readmitted patients (mean 77.1 vs. 67.4 in hospital). |
| **Harris et al., 2005** | SF-36 | No significant difference. |
| **S. Shepperd et al., 1998** | EQ-5D | No significant difference between the two groups |
| **Soleto et al. 2013** | Barthel Index | Both groups improved from initial scores; HaH group maintained higher scores at 12 months |
| **Soleto et al. 2013** | (MLHFQ) | HaH group improved significantly at 6 months (p=0.001) ; no significant difference at 12 months |
| **Soleto et al. 2013** | EQ-5 | HaH group showed significant improvement at discharge (p<0.001) and maintained improvement at 12 months (p=0.036) |
| **Soleto et al. 2013** | SF-36 physical health | HaH group showed significant improvement at 6 months (p=0.001); no significant difference at 12 months |
| **Ricauda et al. 2008** | Activities of Daily Living Scale | No significant difference |
| **Ricauda et al. 2008** | Canadian Neurological Scale | No significant difference |

*Abbreviations: NSD = No significant difference; LoS = Length of stay; QALYs = Quality-adjusted life years; VAS = Visual Analogue Scale;* MLHFQ= *Minnesota Living with Heart Failure Questionnaire.*

## List of supplementary figures

| **Figure S1** | **Publication Bias Assessment of Readmission at 3 Months Funnel Plot** |
| --- | --- |
| **Figure S2** | **Pooled Odds Ratios of Mortality at 1, 2, 3, 6, and 12 months** |
| **Figure S3** | **Pooled Odds Ratios of Readmission at 1, 2, 3, 6, and 12 months** |
| **Figure S4** | **Forest plot of emergency attendance at 3 months across different studies comparing hospital care to home care** |
| **Figure S5** | **Forest plot of patient satisfaction at the end of treatment** |


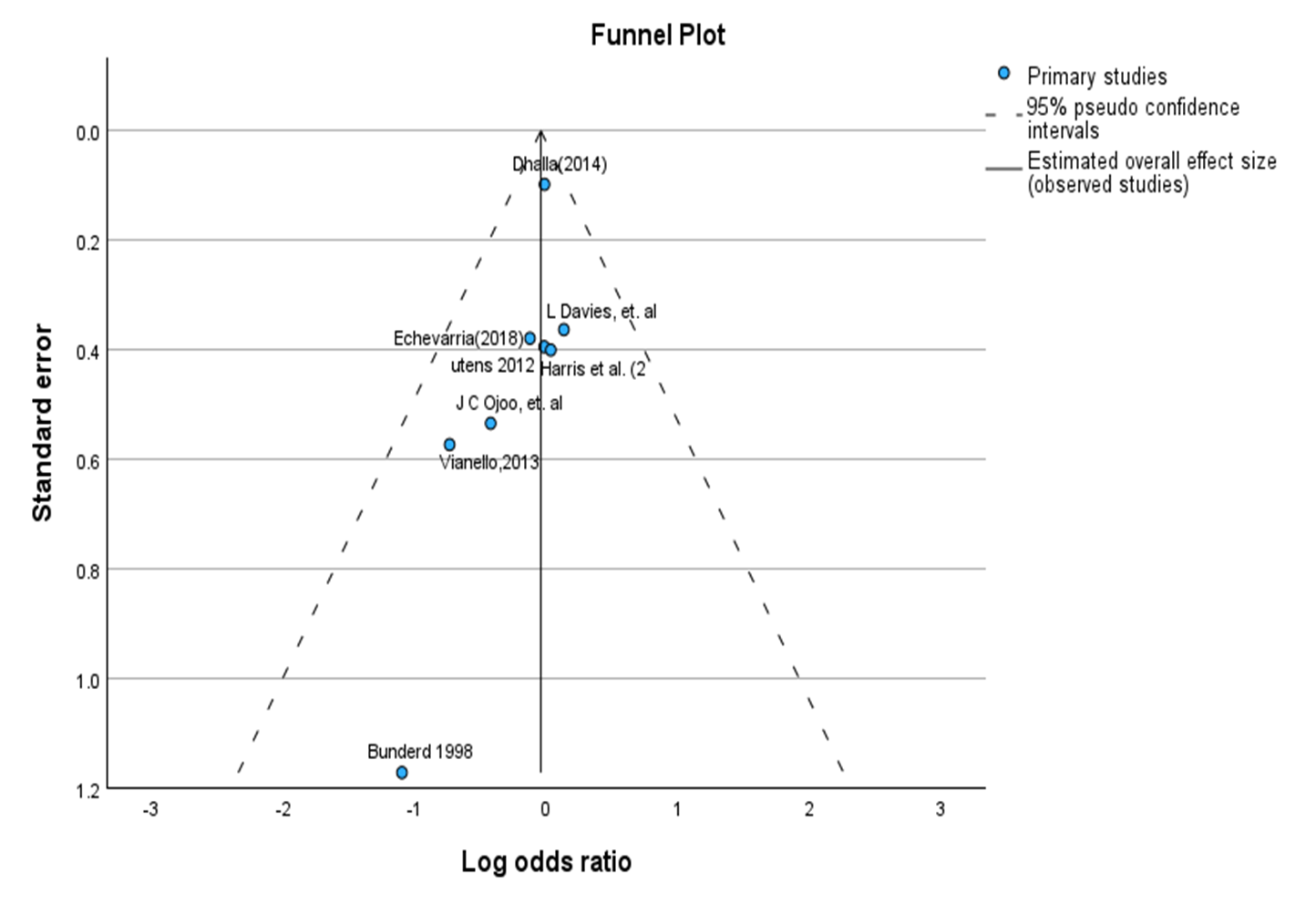
**Figure S1. Publication Bias Assessment of Readmission at 3 Months Funnel Plot**


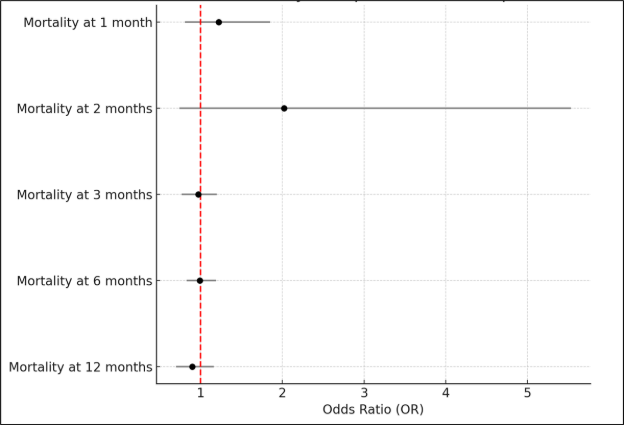
**Figure S2. Pooled Odds Ratios of Mortality at 1, 2, 3, 6, and 12 months**


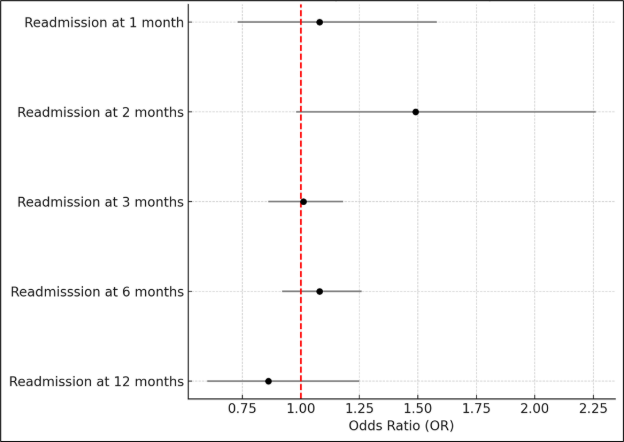


**Figure S3. Pooled Odds Ratios of Readmission at 1, 2, 3, 6, and 12 months**


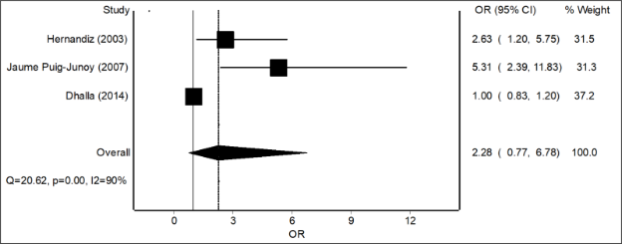


**Figure S4. The forest plot of emergency attendance at 3 months across different studies comparing hospital care to home care**


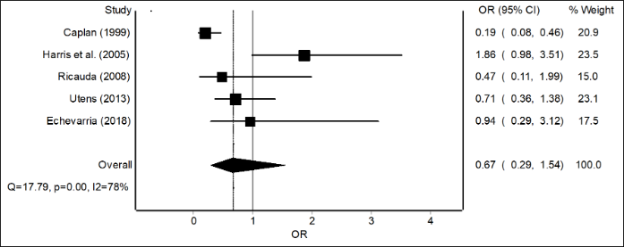


**Figure S5. Forest plot of patient satisfaction at the end of treatment**
